# Allyl Aryl Ether Cleavage by *Blautia* sp. MRG-PMF1 Cocorrinoid *O*-Demethylase

**DOI:** 10.1128/spectrum.03305-22

**Published:** 2022-10-05

**Authors:** Huynh Thi Ngoc Mi, Santipap Chaiyasarn, Bekir Engin Eser, Steven R. Susanto Tan, Supawadee Burapan, Jaehong Han

**Affiliations:** a Metalloenzyme Research Group and Department of Plant Science and Technology, Chung-Ang University, Anseong, Republic of Korea; b Department of Engineering, Aarhus University, Aarhus, Denmark; University of Minnesota

**Keywords:** allyl aryl ether cleavage, biotransformation, *Blautia* sp. MRG-PMF1, cocorrinoid, *O*-demethylase, mechanism, S_N_2′ reaction

## Abstract

Coabalamin-dependent *O*-demethylase in *Blautia* sp. strain MRG-PMF1 was found to catalyze the unprecedented allyl aryl ether cleavage reaction. To expand the potential biotechnological applications, the reaction mechanism of the allyl aryl ether C-O bond cleavage, proposed to utilize the reactive Co(I) supernucleophile species, was studied further from the anaerobic whole-cell biotransformation. Various allyl naphthyl ether derivatives were reacted with *Blautia* sp. MRG-PMF1 *O*-demethylase, and stereoisomers of allyl naphthyl ethers, including prenyl and but-2-enyl naphthyl ethers, were converted to the corresponding naphthol in a stereoselective manner. The allyl aryl ether cleavage reaction was regioselective, and 2-naphthyl ethers were converted faster than the corresponding 1-naphthyl ethers. However, MRG-PMF1 cocorrinoid *O*-demethylase was not able to convert (2-methylallyl) naphthyl ether substrates, and the conversion of propargyl naphthyl ether was extremely slow. From the results, it was proposed that the allyl ether cleavage reaction follows the nucleophilic conjugate substitution (S_N_2′) mechanism. The reactivity and mechanism of the new allyl ether cleavage reaction by cobalamin-dependent *O*-demethylase would facilitate the application of *Blautia* sp. MRG-PMF1 *O*-demethylase in the area of green biotechnology.

**IMPORTANCE** Biodegradation of environmental pollutants and valorization of biomaterials in a greener way is of great interest. Cobalamin-dependent *O*-demethylase in *Blautia* sp. MRG-PMF1 exclusively involves anaerobic C1 metabolism by cleaving the C-O bond of aromatic methoxy group and also produces various aryl alcohols by metabolizing allyl aryl ether compounds. Whereas methyl ether cleavage reaction is known to follow the S_N_2′ mechanism, the reaction pattern and mechanism of the new allyl ether cleavage reaction by cobalamin-dependent *O*-demethylase have never been studied. For the first time, stereoselectivity and the S_N_2′ mechanism of allyl aryl ether cleavage reaction by *Blautia* sp. MRG-PMF1 *O*-demethylase is reported, and the results would facilitate the application of *Blautia* sp. MRG-PMF1 *O*-demethylase in the area of green biotechnology.

## INTRODUCTION

Cocorrinoid *O*-demethylases exclusively involve the anaerobic C-1 metabolism of various bacteria. For example, aryl methyl ethers, methanol, dimethyl sulfide, and methylamines are demethylated by the Co(I)-corrinoid reactive species. The methyl (CH_3_) group of CH_3_-Co(III)-corrinoid is then transferred to tetrahydrofolate or other methyl acceptors to initiate C-1 metabolism (1–6). The *O*-demethylation reaction, especially those of methyl aryl ethers, is valuable for various biotechnology applications. Thus, cocorrinoid *O*-demethylase is considered a promising tool for the lignin valorization (7–10) and bioremediation (11), as well as for the tailoring and protection/deprotection of phenolic compounds (7, 12–14). Lignin, the most abundant source of aromatic polymers on earth, is made up of phenyl methyl ether building blocks (15). Lignin degradation initially leads to aromatic monomers and oligomers that can be utilized as a carbon source through microbial catabolic pathways, in which oxygenase-catalyzed ring opening requires the presence of −OH groups (catechols) rather than −OCH_3_ groups, making demethylation necessary for oxidative assimilation (16). Thus, *O*-demethylation of lignin is important for carbon cycling. Similarly, precursors obtained from lignin require conversion of their −OCH_3_ groups into reactive −OH groups for valorization into value-added products, such as plastic monomers and pharmaceutical building blocks (17). However, *O*-demethylation by chemical means is challenging, requiring harsh reaction conditions, and is often nonselective (7). Therefore, an efficient enzymatic route is desired, and cocorrinoid *O*-demethylase presents such an ecofriendly path under mild conditions. Recently, cocorrinoid *O*-demethylase was also used to construct a reversible methyl transfer shuttle to demethylate a variety of phenyl methyl ethers in the presence of a convenient methyl acceptor, demonstrating the enzyme’s great potential in synthetic applications, such as deprotection reactions, to unmask hydroxyl groups, as well as tailoring of natural products (7, 13, 14).

The same enzyme also plays an important role in human health by metabolizing dietary natural products (18–20). For example, partial demethylation of flavonoids and curcuminoids produce bioactive metabolites exhibiting anti-inflammatory and anti-Alzheimer activity (21, 22). Thus, various partially demethylated polymethoxyflavone derivatives can be enzymatically synthesized by *O*-demethylase for the investigation of their bioactivities in preclinical and clinical studies.

Whereas cocorrinoid-dependent *O*-demethylases, metabolizing a broad spectrum of methylated substrates, have been reported from various microorganisms (23), the reaction mechanism of the C-O bond cleavage by this enzyme has not been thoroughly studied. Chemically, the initial reaction step of demethylation that produces CH_3_-Co(III)-corrinoid species is believed to follow an S_N_2-type mechanism according to the theoretical and synthetic model complex studies, even though both radical and ionic reactions are feasible (24, 25). On the other hand, the nucleophilicity of the reactive Co(I) species is strong enough to attack the methyl group of methyl aryl ether, and the reactive Co(I) species is often called “supernucleophile” (26).

The reactivity of the flavone *O*-demethylase, recently reported from *Blautia* sp. strain MRG-PMF1 (27), was unique in that it could demethylate relatively large substrates such as curcumin and quercetin pentamethyl ether (21, 22). Beforehand, biotransformation of most *O*-demethylases was limited to small substrates, such as guaiacol, which hindered biotechnological applications of *O*-demethylase. Moreover, this cocorrinoid *O*-demethylase can successively demethylate bulky polymethoxyflavone molecules at various positions on the aromatic rings (21), whereas most other *O*-demethylases are strict in their regioselectivity (7, 28). Thus, the cocorrinoid *O*-demethylase system, with a broad substrate spectrum and regiodiversity (demethylation of various −OCH_3_ positions), is appealing for biotechnology and medical applications.

The extraordinary reactivity of cocorrinoid *O*-demethylase of *Blautia* sp. MRG-PMF1 further exemplified the cleavage of prenyl ether group of isoimperatorin (29). Chemical cleavage of allyl aryl ether is also difficult and often requires strong Lewis acids, such as metal ions, to localize the electron distribution around the oxygen atom of the ether group (30). The related allyl aryl ether cleavage reaction is only available from the chemical model system, which utilizes the photoreduced vitamin B_12_ catalyst (31).

**FIG 1:**
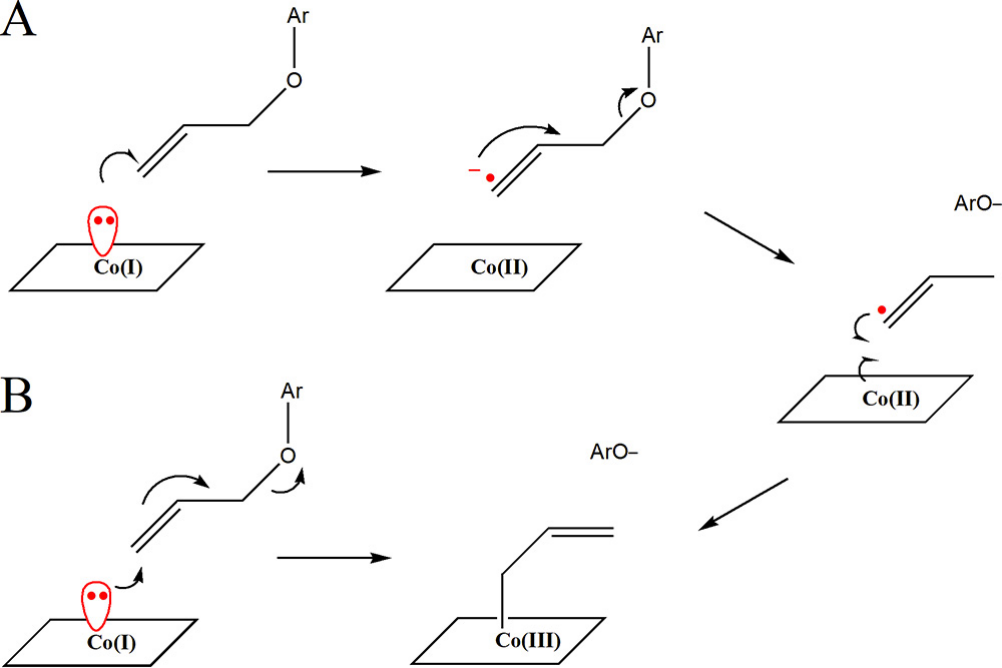
Two possible reaction mechanisms of allyl aryl ether cleavage by the Co(I) reactive species of cocorrinoid *O*-demethylase. (A) The electron transfer mechanism involves a single electron transfer from Co(I)-corrinoid to the substrate, resulting in Co(II)-corrinoid and allyl aryl ether anion radical. The anion radical species rearranges to the corresponding alcohol and allylic radical species that bind to Co(II)-corrinoid to form allyl-Co(III)-corrinoid. (B) For the nucleophilic conjugate substitution mechanism, Co(I) nucleophile attacks the terminal trigonal unsaturated (*sp*^2^) carbon of allyl ether, which spontaneously results in the formation of products.

**FIG 2:**
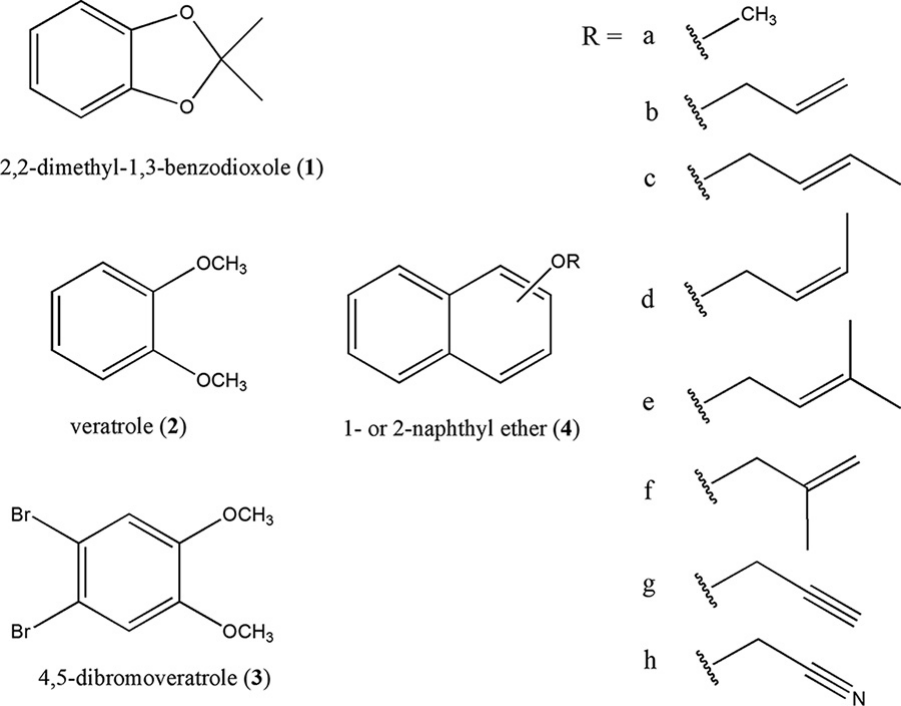
Substrates used for the study. For naphthyl ether (substrate 4), the position of naphthyl ether group was designated by the number. For example, allyl 2-naphthyl ether was labeled 4b2, and *cis*-but-2-enyl 1-naphthyl ether was labeled 4d1.

The new activity of *O*-demethylase appears to utilize the same Co(I) reactive species, and both electron transfer and nucleophilic substitution reactions by Co(I)-corrinoid are possible for the mechanism of the allyl aryl ether cleavage reaction (Fig. 1). Depending on the reaction pathways, different biotransformation products, as well as intermediates and transition-state complexes, are expected to form when different allyl aryl ether substrates are reacted (31). Therefore, various aryl ether compounds were synthesized and biotransformed by *Blautia* sp. MRG-PMF1 to study the reactivity and reaction mechanism of allyl aryl ether cleavage by cocorrinoid *O*-demethylase (Fig. 2).

## RESULTS

### Synthesis of the substrates.

All of the substrates used in this study were prepared according to the methods described in the literature, and details of purification with flash column chromatography by applying ethyl acetate gradient in hexane eluent of syntheses and characterizations are available in Text S1 and S10 in the supplemental material. For naphthyl ether (Fig. 2, substrate 4) substrates, both allyl 1- and 2-naphthyl ether derivatives were synthesized, and the position of the ether group was designated by the number. For example, allyl 1-naphthyl ether and allyl 2-naphthyl ether were designated ether 4b1 and ether 4b2, respectively (Fig. 2).

**FIG 3:**
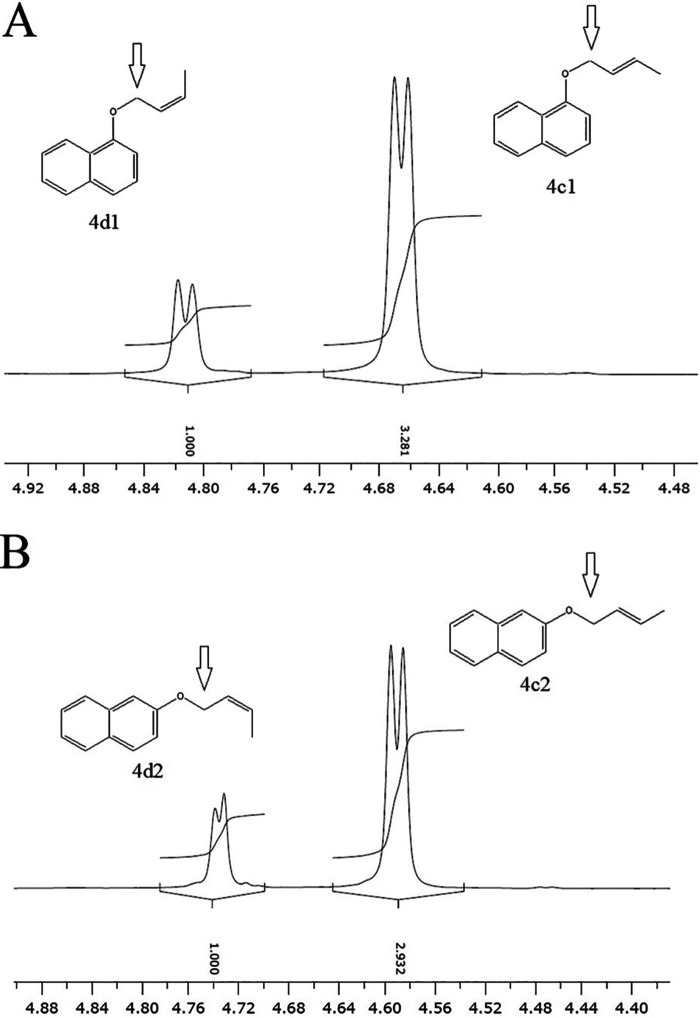
The methylenyl proton peaks of *trans*-but-2-enyl 1-naphthyl ether (4c1) and *cis*-but-2-enyl 1-naphthyl ether (4d1) (A) and *trans*-but-2-enyl 2-naphthyl ether (4c2) and *cis*-but-2-enyl 2-naphthyl ether (4d2) (B) in the ^1^H-NMR spectra. For both, *trans*-isomers were found more than *cis*-isomers by about 3 times.

It should be mentioned that a mixture of *cis*- and *trans*-crotyl chloride was used for the synthesis of but-2-enyl 1-naphthyl ethers (4c and 4d). The isomeric mixture of *trans*-but-2-enyl 1-naphthyl ether (4c1) and *cis*-but-2-enyl 1-naphthyl ether (4d1) was purified by column chromatography from the reaction between crotyl chloride and 1-naphthol. Likewise, the isomeric mixture of *trans*-but-2-enyl 2-naphthyl ether (4c2) and *cis*-but-2-enyl 2-naphthyl ether (4d2) was prepared from the reaction between crotyl chloride and 2-naphthol. Because *trans*-isomers of ethers 4c1 and 4c2 were abundant, the *trans*- and *cis*-isomeric mixture products were not further isolated. Both geometric isomers were easily identified from the different chemical shifts and coupling constants of the methylene protons by nuclear magnetic resonance (NMR) spectroscopy, and the ratios of *trans*- and *cis*-isomers in both mixtures were determined by integration of the peaks (Fig. 3). The ratios of 3.3:1 and 2.9:1 were found for ethers 4c1/4d1 and ethers 4c2/4d2, respectively.

### Biotransformation of synthetic aryl methyl ether compounds.

*Blautia* sp. MRG-PMF1 *O*-methylase is known to convert small aryl methyl ethers such as syringic acid and vanillic acid. It also demethylated methyl 1- and 2-naphthyl ethers (4a1 and 4a2) completely within a day to the corresponding naphthol products (Text S3). However, it could not metabolize 2,2-dimethyl-1,3-benzodioxole (Fig. 2, substrate 1) or other long-chain alkyl aryl ethers, such as ethyl, propyl, and butyl aryl ether (21). Veratrole (Fig. 2, substrate 2) and 4,5-dibromovertarole (Fig. 2, substrate 3) were transformed to the corresponding catechol products, but the bromo group of 4,5-dibromovertarole (Fig. 2, substrate 3) was not metabolized at all (Text S2). Therefore, it was concluded that MRG-PMF1 *O*-demethylase does not cleave the carbon-halogen bond. Biotransformation of these small synthetic organic compounds shows a possibility of *Blautia* sp. MRG-PMF1 *O*-demethylase in the application of organic synthesis and lignin valorization.

### Biotransformation of synthetic allyl aryl ether compounds.

**FIG 4:**
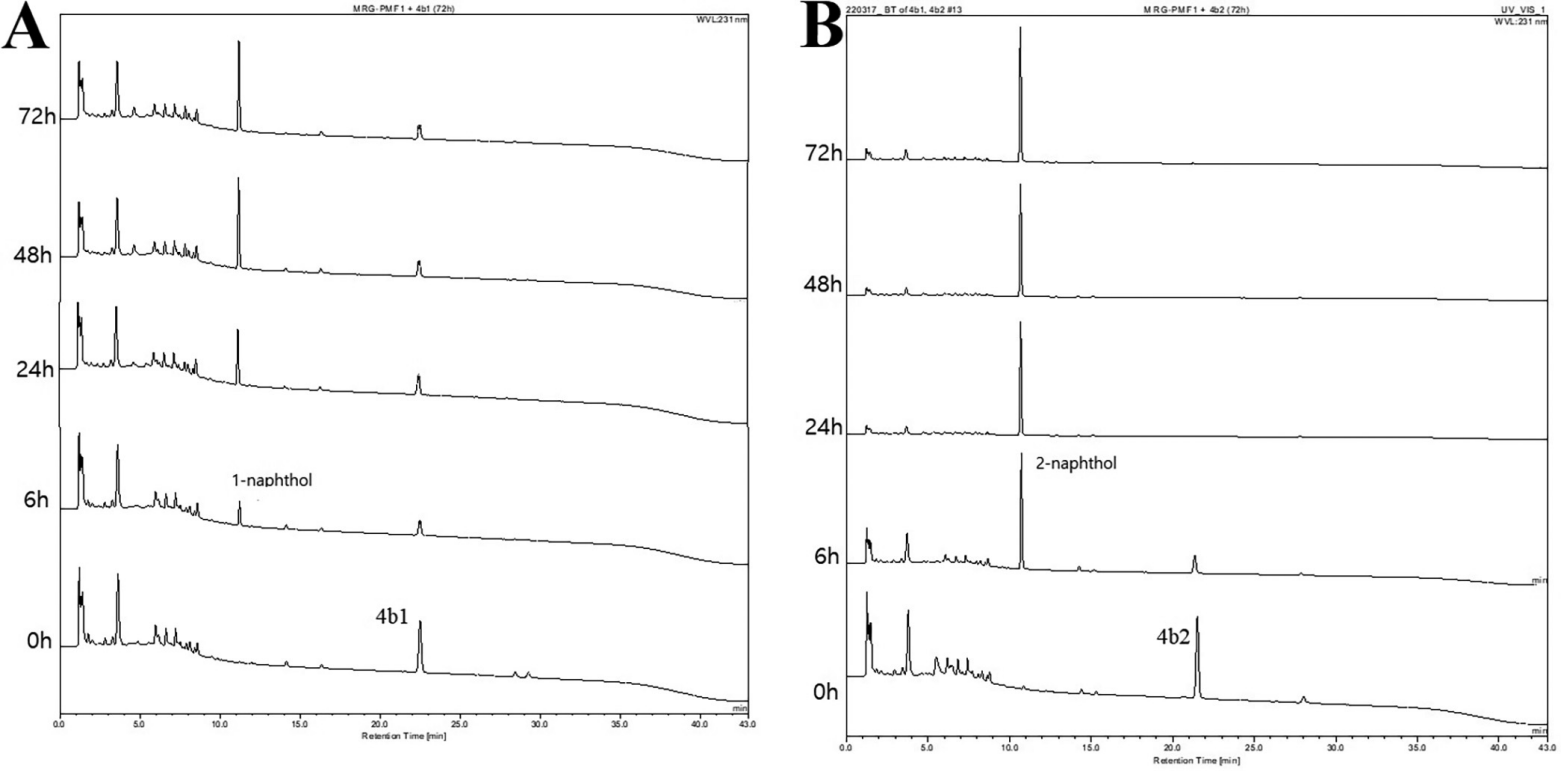
Biotransformation of allyl naphthyl ethers was monitored by HPLC. Conversion of 4b1 (A) and 4b2 (B) was monitored at the wavelengths of 231 nm and 225 nm, respectively.

After investigation of methyl aryl ether cleavage reactions, allyl naphthyl ethers were used as the substrates to check the reactivity of allyl aryl ether cleavage by *Blautia* sp. MRG-PMF1 *O*-demethylase. Among the reacted substrates, allyl naphthyl ethers (4b), *trans*-but-2-enyl naphthyl ether (4c), *cis*-but-2-enyl naphthyl ether (4d), and prenyl naphthyl ether (4e) in Fig. 2 were found to be converted to the corresponding naphthol products (Fig. 4 and Text S4 to S6). For 4b, 95% of allyl 2-naphthyl ether (4b2) was metabolized within 24 h, whereas allyl 1-naphtyl ether (4b1) was converted by 70% even after 72 h. The conversion of but-2-enyl naphthyl ether substrates (4c and 4d) is of interest since it is reported that the photogenerated Co(I) catalytic species cannot convert these substrates (31).

**FIG 5:**
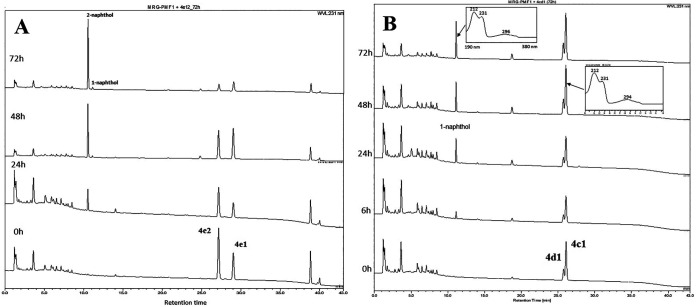
Biotransformation of a mixture of prenyl 1-naphthyl ether (4e1) and prenyl 2-naphthyl ether (4e2) (A) and a mixture of *trans*-but-2-enyl 1-naphthyl ether (4c1) and *cis*-but-2-enyl 1-naphthyl ether (4d1) (B) were monitored by HPLC at the wavelength of 231 nm.

The results obtained from biotransformations of ethers 4a, 4b, 4c, 4d, and 4e demonstrated the characteristics of *Blautia* sp. MRG-PMF1 *O*-demethylase-catalyzed reactions. First, cleavage of the methyl naphthyl ether bond (4a) was faster than that of the allyl naphthyl ether bond (4b to 4e). Second, 2-naphthyl ether substrates reacted faster than the corresponding 1-naphthyl ether substrates. For example, similar to ether 4b, the conversion of *cis*- and *trans*-but-2-enyl 2-naphthyl ethers (4c2 and 4d2) was completed in 24 h, while the isomers of *cis*- and *trans*-but-2-enyl 1-naphthyl ethers (4c1 and 4d1) were partially metabolized even in 72 h. The regioselectivity for allyl 2-naphthyl ethers was consistent and prenyl 2-naphthyl ether (4e2) was also converted faster than prenyl 1-naphthyl ether (4e1). It was evident from the biotransformation of the prenyl naphthyl ether (4e) mixture (Fig. 5A). 2-Naphthol was the early product produced by *O*-demethylase when both regioisomers were reacted. Third, the reaction rates were found in the order of ethers 4b > 4c > 4d > 4e, suggesting that the reaction is controlled by steric hindrance of substrates. The cleavage of prenyl naphthyl ethers 4e1 and 4e2 is of significance in that the prenylated compounds are emerging natural products due to their unique bioactivity. It is especially noteworthy that *trans*-but-2-enyl naphthyl ether (4c1) was converted faster than *cis*-but-2-enyl naphthyl ether (4d1) (Fig. 5B). Therefore, it was concluded that *Blautia* sp. MRG-PMF1 *O*-demethylase catalyzed allyl aryl ether cleavage reaction in a regio- and stereoselective manner.

However, it was surprising that both regioisomers of (2-methylallyl) 1-naphthyl ether (4f1) and (2-methylallyl) 2-naphthyl ether (4f2) were almost inert to the allyl aryl ether cleavage reaction by *Blautia* sp. MRG-PMF1 *O*-demethylase (Text S7). Regardless of the structural similarity to the other ally naphthyl ether and prolonged reaction times, only traces of 1- or 2-naphthol production were observed from the reaction mixtures.

To further extend the reactivity study, propargyl 2-naphthyl ether (4g2) and two cyanomethyl naphthyl ether isomers (4h1 and 4h2) were reacted with *Blautia* sp. MRG-PMF1 *O*-demethylase. The results from the triple-bond derivatives were similar to those from ether 4f. Only traces of the corresponding naphthol products were observed (Text S8 and S9).

### Mechanism of allyl aryl ether cleavage by cocorrinoid-dependent *O*-demethylase.

**FIG 6:**
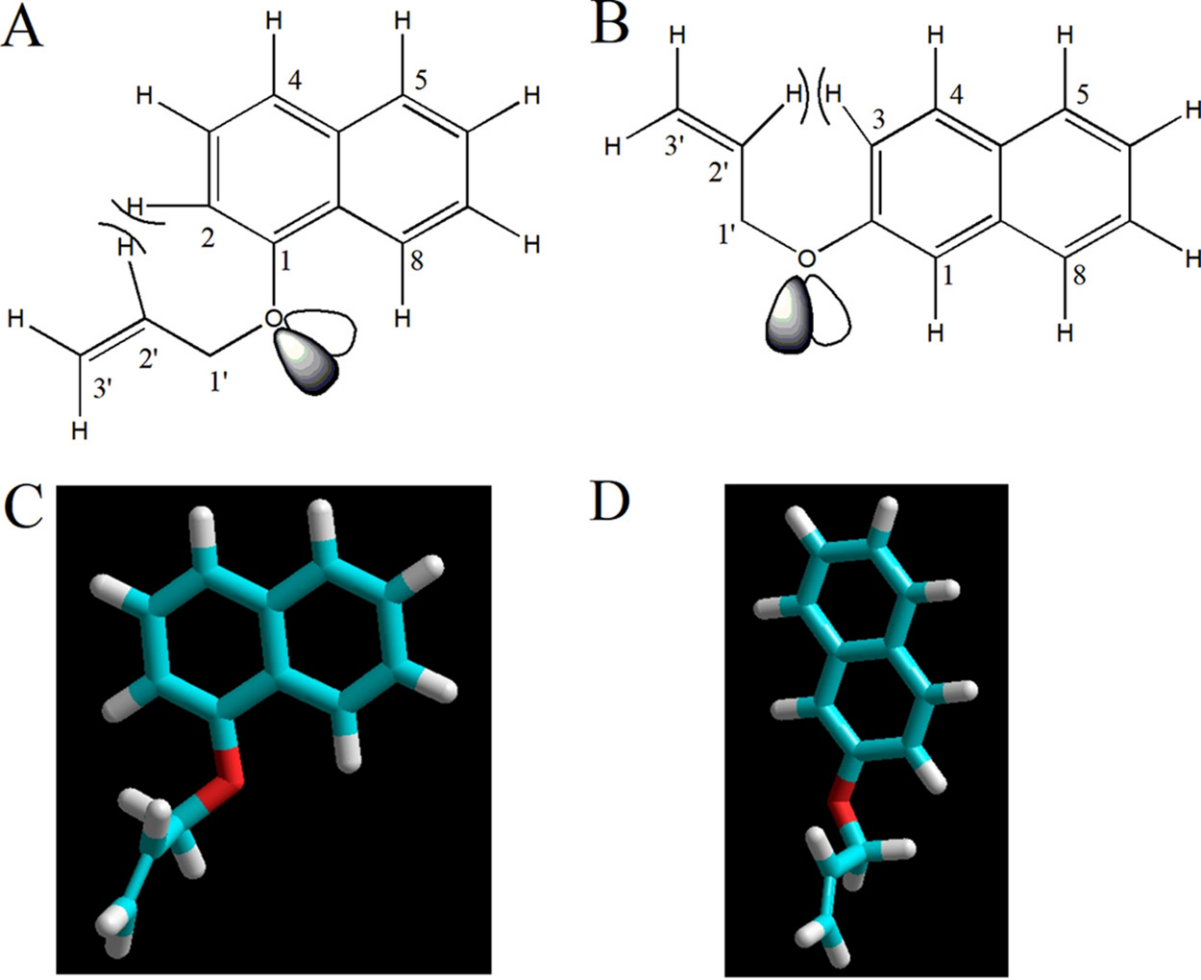
Molecular structures of ally naphthyl ether substrates. Allyl 1-naphthyl ether (A) and allyl 2-naphthyl ether (B) substrates have limited rotation of the C-1-O-C-1′-C-2′ torsional angle due to the steric hindrance. Geometry optimized structures of allyl 1-naphthyl ether (4b1) (C) and allyl 2-naphthyl ether (4b2) (D) were obtained from semiempirical PM3 calculations.

The reaction pattern of *Blautia* sp. MRG-PMF1 is summarized in Table 1. It was intriguing that all the allyl naphthyl ether substrates, except 2-methylallyl naphthyl ethers (4f1 and 4f2), were metabolized by *Blautia* sp. MRG-PMF1 *O*-demethylase. Hence, computational chemistry of the substrates was performed to search the energy-minimized molecular structure and stable conformers. First, the molecular structures of allyl 1-naphthyl ether substrates in Fig. 2 were calculated. Conformation of allyl naphthyl ethers (4b1 and 4b2) is mainly fixed due to oxygen 8-H interaction, so the stable conformation can be searched by changing the torsional angle of C-1-O-C-1′-C-2′ (Fig. 6). The torsional angle of 0° showed the highest energy due to the steric hindrance between H-2 and H-2′, but the rotation of the O-C-1′ bond was accessible at room temperature with the C-1-O-C-1′-C-2′ torsional angle between 60° and 320° by less than 10 kcal/mol energy barrier (Text S11). The potential energy minimum of ethers b1 was found with the C-1-O-C-1′-C-2′ torsional angle of 277°. With this conformer, the potential energy of the O-C-1′-C-2′-C-3′ torsional angle was calculated, and the highest rotational energy barrier was lower than 2.0 kcal/mol. It was evident that the rotation of the C-1′-C-2′ bond in the allyloxy group is practically free, and the allyloxy group is very flexible at room temperature. The same results were obtained from all the other allyl 1-naphthyl ether substrates from ethers 4c1 to 4f1 (Text S11).

**TABLE 1:**
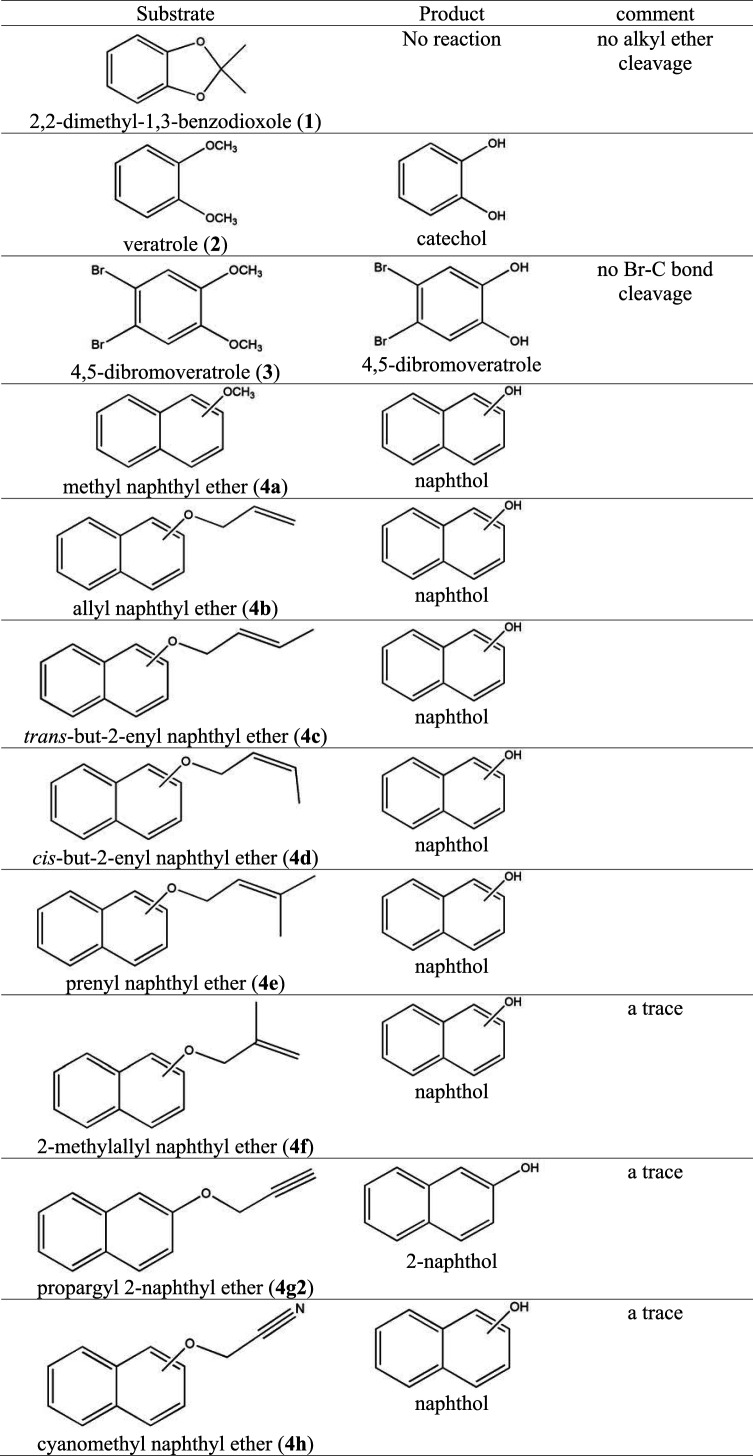
Biotransformation of aryl ethers by *Blautia* sp. MRG-PMF1 *O*-demethylase

## DISCUSSION

The results obtained from biotransformations of naphthyl ethers demonstrated the characteristics of *Blautia* sp. MRG-PMF1 *O*-demethylase-catalyzed reactions. Cleavage of the methyl naphthyl ether bond (4a) was faster than that of the allyl naphthyl ether bond (4b to 4e), and 2-naphthyl ether substrates reacted faster than the corresponding 1-naphthyl ether substrates. Furthermore, the reaction rates of allyl aryl ether cleavage were found in the order of ethers 4b > 4c > 4d > 4e, suggesting that the reaction is controlled by steric hindrance of substrates. Therefore, it was concluded that *Blautia* sp. MRG-PMF1 *O*-demethylase catalyzed the allyl aryl ether cleavage reaction in a regio- and stereoselective manner.

**FIG 7:**
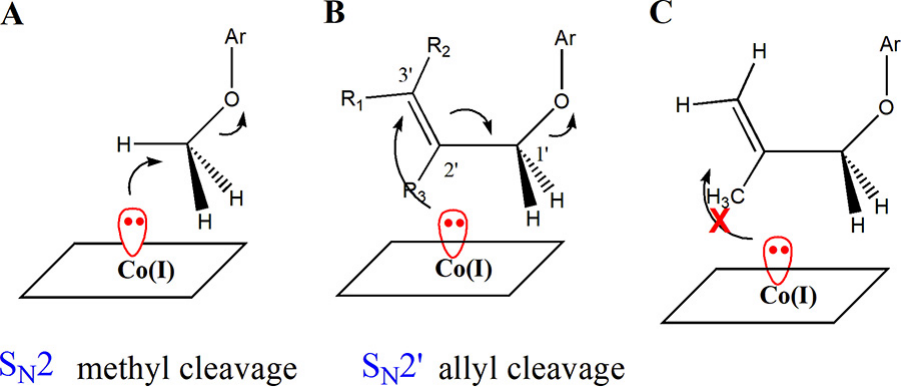
Proposed reaction mechanisms of C-O bond cleavage reaction by cocorrinoid *O*-demethylase. The nucleophilic substitution S_N_2 mechanism (A) and nucleophilic conjugate substitution S_N_2′ mechanism (B) for the cleavage of C-O bond of aryl methyl ether and allyl aryl ether, respectively, were proposed. (C) The ally aryl ether bond of 1-methylallyl naphthyl ethers, 4f1 and 4f2, cannot be cleaved by cocorrinoid *O*-demethylase due to the incapability of the substrates to form the productive conformation at the active site.

From the computational chemistry, it was found that allyloxy functional groups of the aryl ether substrates in the study were very flexible, and energy barriers for the conformational changes were insignificant. However, considering the stereospecificity of the S_N_2- and S_N_2′-type reactions and the structure of cocorrinoid, the substrates were expected to be positioned as shown in Fig. 7A. The Co(I) reactive species should attack methyl carbon from the opposite side of the aryloxy-leaving group to form the CH_3_-Co(III) product. Similarly, the same reactive Co(I) should attack one side of the C-3′ trigonal plane. Whereas allyl aryl ether substrates 4b1 and 4b2 would not have much steric hindrance in the active site, bulkier substituents of the allyl group could not allow the reactive conformation of the substrates. For example, a methyl substitution for R_3_ would interfere with the direct attack of the Co(I) reactive species, while methylation of R_1_ and R_2_ would not interfere with the reaction (Fig. 7A). In the active site, the allylic group of ethers 4f1 and 4f2 cannot be oriented to the productive conformation due to the steric hindrance of the C-1′-CH_3_ group to the corrin ring Co(I)-cobalamin. Therefore, the allylic ether bond of ethers 4f1 and 4f2 cannot be cleaved as shown in Fig. 7B. One of the relevant protein X-ray crystallographic structures of cocorrinoid-dependent enzymes showed that the cofactor protruded from the surface of the protein, probably to access the substrate bound to the substrate binding protein (31). Considering the broad spectrum of substrates catalyzed by *Blautia* sp. MRG-PMF1 *O*-demethylase, we think the substrate binding domain/enzyme of the cocorrinoid *O*-demethylase has a large substrate binding pocket. Even so, the observed reactivity of allyl aryl ether cleavage proposed that it follows a certain reaction mechanism.

The reaction mechanism of CH_3_-O bond cleavage by cocorrinoid-dependent *O*-demethylase is established to follow the S_N_2 mechanism. From the reactivity study of *O*-demethylase with various allyl naphthyl ethers (see above), the allyl ether cleavage reaction appears to follow the S_N_2′ reaction mechanism. As shown in Fig. 7, both S_N_2 and S_N_2′ mechanisms require the antiperiplanar arrangement of nucleophile [Co(I)-cobalamin] and leaving group (O-Ar), the stereoelectronic control. The proposed S_N_2′ mechanism can also explain the extremely slow conversion of propargyl 2-naphthyl ether (4g2) and cyanomethyl 2-naphthyl ether (4h2) by *O*-demethylase because the functional groups are very flexible in the active site, so the leaving group cannot be antiperiplanar.

In this study, it was found that the C-O bond cleavage of allyl aryl ether is stereoselective and follows the S_N_2′ reaction mechanism. An earlier study of allyl aryl ether cleavage reactions performed by photoinduced vitamin B_12_ complex also proposed the S_N_2′ mechanism, consistent with our enzymatic system (32). Although cocorrinoid *O*-demethylases from anaerobic bacteria have been identified earlier, substrate scope and mechanistic studies on the enzymes involved have been scarce. Expansion of the regio- and stereoselective reactivity of cocorrinoid *O*-demethylase toward allyl naphthyl ethers, in addition to aryl methyl ethers, is an important step for broadening the use of this enzyme in biotechnology, medicine, and synthetic applications. In addition to expanding our knowledge on the different strategies of ether bond cleavage in nature, the mechanistic information presented here will help efforts to further optimize and engineer *O*-demethylases for future applications.

## MATERIALS AND METHODS

**Biotransformation of allyl naphthyl ethers by Blautia sp. MRG-PMF1.** All of the experimental procedures for the biotransformation, including bacterial culture in deoxygenated Gifu anaerobic medium, were performed under anaerobic conditions (5% CO_2_, 10% H_2_, 85% N_2_) at 35°C, except for the metabolite analysis. The anaerobic culture of *Blautia* sp. MRG-PMF1 was grown at 35°C until it reached an optical density at 600 nm (OD_600_) of ~0.6. We added 100 μL of the substrate (10 mM in dimethylformamide [DMF]) to the bacterial culture (10 mL). Aliquots (3 mL) of the biotransformation mixture were transferred to test tubes after scheduled reaction times. The allocated culture was added with 3 mL of ethyl acetate, vortexed for 1 min, and centrifuged at 10,770 × *g* for 10 min to collect the organic phase. The extraction was repeated twice, and the combined organic fraction was dried under vacuum. The dried residue was dissolved in MeOH (300 μL) and filtered through a 0.2-μm polytetrafluoroethylene (PTFE) filter (Advantec, Japan) for the chromatography analysis.

**HPLC analysis of biotransformation products.** A Dionex UltiMate 3000 ultrahigh-performance liquid chromatography (UHPLC) system (Thermo Fisher Scientific, USA) with a diode array detector (DAD), equipped with a Kinetex C_18_ column (1.7 μm particle size, 100 by 2.1 mm inner diameter [i.d.]; Phenomenex, USA) was used for HPLC analysis. Program setup, data collection, and analysis were conducted using Chromeleon chromatography data system (CDS) software version 6.80 (Thermo Fisher Scientific, USA). The detector was set to record at 231 nm for allyl 1-naphthyl ether biotransformation products analysis or at 225 nm for allyl 2-naphthyl ether biotransformation product analysis, simultaneously with UV spectrum monitoring in the range of 190 to 380 nm. Chromatographic conditions were set as follows: injection volume was 1 μL, the flow rate was 0.2 mL/min, and the column temperature was 25°C. HPLC eluents consist of 0.1% acetic acid (vol/vol) in water (A) and MeCN (B), and a multistep gradient program was employed. For the analysis of 4a biotransformation products, solution B was started at 10%, increased to 40% for 2 min and 90% for 13 min, and was held for 10 min. For 4b, solution B was started at 10%, increased to 40% for 2 min and 90% for 13 min, and was held for 5 min. For the analysis of 4c and 4d biotransformation products, solution B was started at 10%, increased to 40% for 2 min, 70% for 28 min, and 90% for 5 min, and was held for 5 min. For 4g biotransformation, solution B was started at 10%, increased to 30% for 2 min, 50% for 20 min, and 90% for 5 min, and was held for 5 min.

### Geometry optimization of substrates.

Molecular structures of allyl naphthyl ethers were generated by HyperChem (HyperChem 8.0 for Windows; Hypercube, Gainesville, FL, USA). Geometry optimization of the built models was performed using the PM3 semiempirical method, and Polak-Ribiere was chosen as the minimization algorithm (33). The rotational energy barrier of the torsional angles was calculated by potential energy calculation with the optimized structures.

## Supplementary Material

Reviewer comments
