# Correction for Mi et al., “Allyl Aryl Ether Cleavage by *Blautia* sp. MRG-PMF1 Cocorrinoid *O*-Demethylase”

**DOI:** 10.1128/spectrum.00072-23

**Published:** 2023-01-30

**Authors:** Huynh Thi Ngoc Mi, Santipap Chaiyasarn, Bekir Engin Eser, Steven R. Susanto Tan, Supawadee Burapan, Jaehong Han

## AUTHOR CORRECTION

Volume 10, no. 5, e03305-22, 2022, https://doi.org/10.1128/spectrum.03305-22. The first paragraph of the Acknowledgments section should read as follows: “This research was supported by the Chung-Ang University Research Grants in 2019 and the Basic Science Research Program through the National Research Foundation of Korea (NRF) funded by the Ministry of Education (NRF-2021R1A2C2007712).”

