# Supplementary material for: Allyl Aryl Ether Cleavage by Blautia sp. MRG-PMF1 Cocorrinoid O-Demethylase
Source: Microbiol Spectr. 2022 Oct 5;10(5):e03305-22. doi: 10.1128/spectrum.03305-22 (PMC9602652; doi:10.1128/spectrum.03305-22)

Supplemental Material to

Allyl Aryl Ether Cleavage by *Blautia* sp. MRG-PMF1 Co-Corrinoid *O*-Demethylase

Huynh Thi Ngoc Mi,[†] Santipap Chaiyasarn,[†] Bekir Engin Eser, [‡] Tan Steven R. Susanto,[†]

Supawadee Burapan,[†,§] and Jaehong Han*,[†]

S1. Preparation of substrates

S2. Biotransformation of veratrole (**2**) and 4,5-dibromovertrole (**3**)

S3. HPLC analysis of **4a** biotransformation

S4. HPLC analysis of **4c2** and **4d2** biotransformation

S5. HPLC analysis of a mixture of but-2-enyl 2-naphthyl ether (**4c** and **4d**) biotransformation

S6. HPLC analysis of **4e** biotransformation

S7. HPLC analysis of **4f** biotransformation

S8. HPLC analysis of **4g** biotransformation

S9. HPLC analysis of **4h** biotransformation

S10. NMR spectra of the compounds

S11. Computational study results

S1. Preparation of methylated substrates

Acetone, anhydrous potassium carbonate, phosphorus pentoxide, ethyl acetate, and diethyl ether were purchase from DAEJUNG Chemicals & Metals Co. (Seoul, Korea). Methyl iodide, crotyl chloride, prenyl bromide, catechol and 1-, 2-naphthol were purchased from ThermoFisher Scientific (Seoul, Korea). Veratrole (**2**) was purchased from Sigma-Aldrich,

For the synthesis of ethers, alkyl halide and aromatic alcohols were reacted in the presence of anhydrous potassium carbonate for overnight. The reaction mixture was washed and organic layer was collected for chromatographic isolation. Hexanes and ethyl acetate were used for elution, and the purity of compound was checked by TLC. Each compound was characterized by NMR spectroscopy. Spinworks 4 S/W was used for spectrum manipulations.[1]

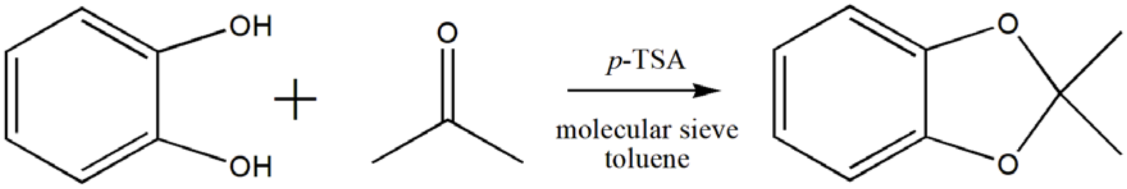

catechol

2,2-dimethyl-1,3-benzodioxole (**1**)

2,2-Dimethyl-1,3-benzodioxole (1)[2] In the presence of catalytic amounts of *p*-toluenesulfonic acid (*p*-TSA) and molecular sieve (10g), catechol (55g) was refluxed in the mixture of dried acetone and toluene (300mL, 1:1) for 12 hours. When the reaction was completed, the reaction mixture was concentrated with rotary evaporator and the residue was dissolved in petroleum

[1] Spinworks 4 by Kirk Marat, University of Manitoba, Canada

[2] Cole ER, Crank G Minh HTH (1980) An improved method for the synthesis of 2,2-disubstituted and 2-monosubstituted 1,3-benzodioxoles. Australian Journal of Chemistry 33, 675-680. https://doi.org/10.1071/CH9800675

ether (200mL) to filter through the Celite bed. The compound **1** was isolated as a clear oil (35g, 47%) after removal of solvent. The compound was detected by 270nm UV on silica gel TLC, but was not visible with I_2 vapor reaction.

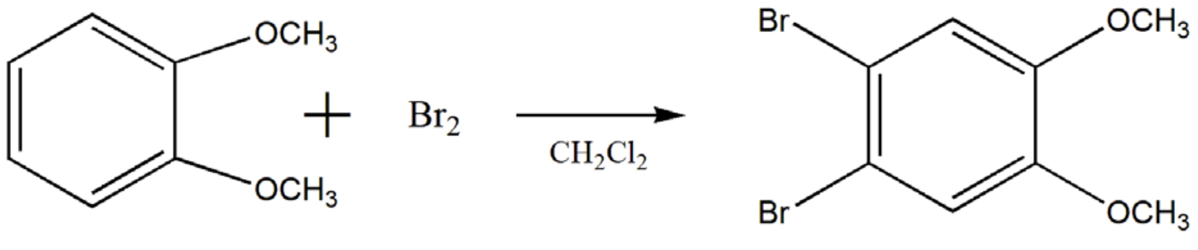

veratrole (**2**)   4,5-dibromoveratrole (**3**)

4,5-Dibromoveratrole (**3**)[3] To a flask containing veratrole (**2**) (2.62 mL, 20 mmol) dichloromethane solution (40 mL), a solution of bromine (2.04 mL, 40 mmol) in 6 mL of dichloromethane was added dropwise. After 20 hours, the reaction mixture was transferred to separatory funnel and washed with saturated sodium metabisulfite solution (50 mL), saturated sodium bicarbonate solution (50 mL), and water twice. The organic layer was dried under the reduced pressure, and the product (4.44g, 75%) was recrystallized in acetone. ^{1}H-NMR ($CDCl_3$, 600 MHz), δ 3.93 (2H, aromatic H), 7.01 (6H, CH_3O).

[3] Wenderski, T., Light, K. M., Ogrin, D., Bott, S. G., & Harlan, C. J. (2004). Pd catalyzed coupling of 1, 2-dibromoarenes and anilines: formation of N, N-diaryl-o-phenylenediamines. Tetrahedron letters, 45(37), 6851-6853. https://doi.org/10.1016/j.tetlet.2004.07.116

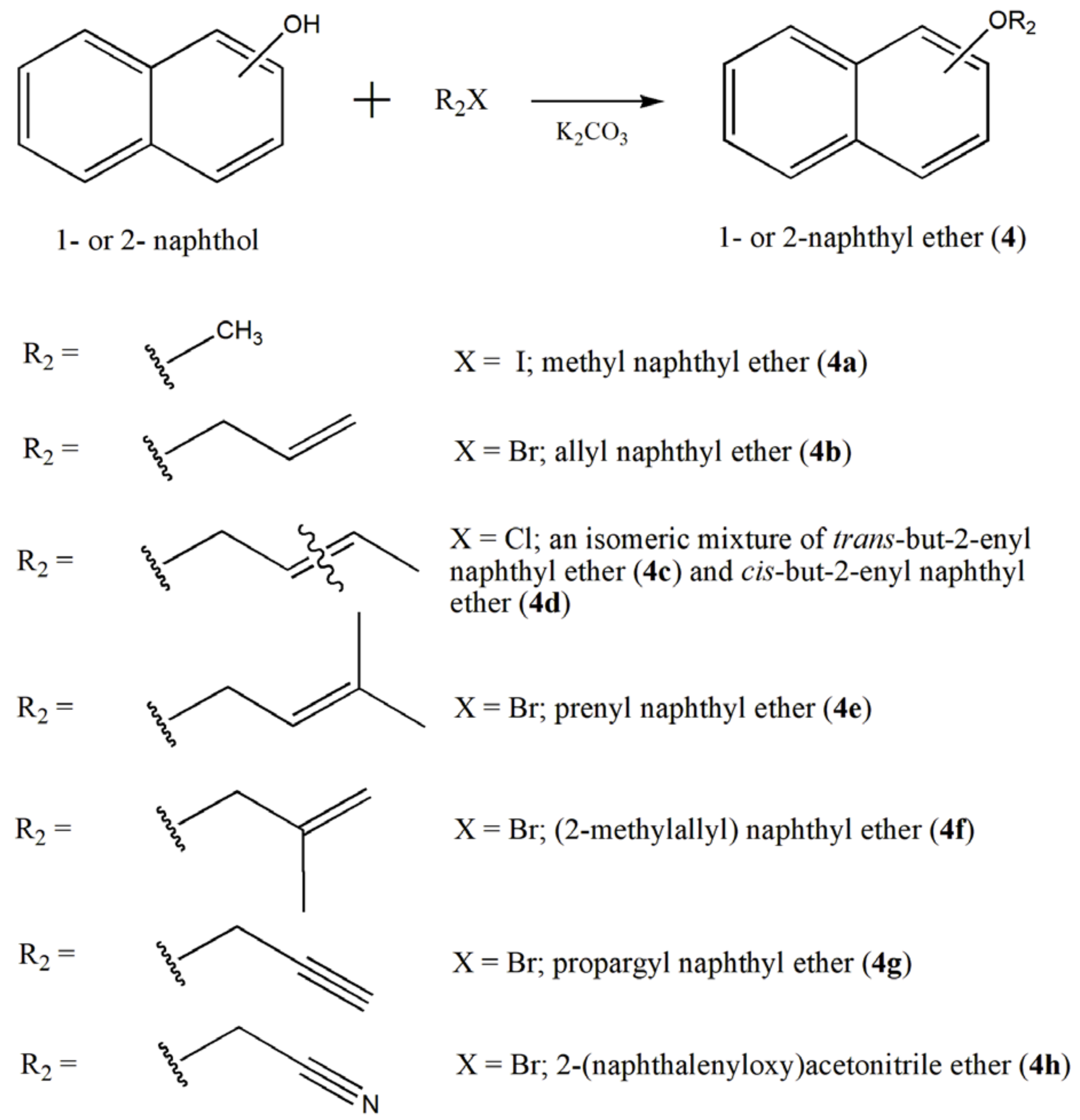

Methyl 1-naphthyl ether (4a1)[4] Methyl iodide (1.0 mL) 2-naphthol (1.45 g), and K_2CO_3 (1.5 g) were added in 20 mL of DMF. The reacted in the presence of anhydrous potassium carbonate. After 12 hrs, the reaction mixture was filtered and the filtrate was extracted by

---

[4] Yoshida, S., Shimizu, K., Uchida, K., Hazama, Y., Igawa, K., Tomooka, K., & Hosoya, T. (2017). Construction of condensed polycyclic aromatic frameworks through intramolecular cycloaddition reactions involving arynes bearing an internal alkyne moiety. Chemistry–A European Journal, 23(61), 15332-15335.

EtOAc (30 mL X 2). The combined organic solution was washed with brine and dried over anhydrous Na_2SO_4. The compound was isolated by silica gel column chromatography. Colorless oil. UV (PDA, H_2O/MeCN, nm) 211, 230(sh).

Methyl 2-naphthyl ether (4a2) prepared similar to **4a1** by using 2-naphthol. White solid. UV (PDA, H_2O/MeCN, nm) 225.

Allyl 1-naphthyl ether (4b1) Allyl bromide and 1-naphthol were reacted. Colorless oil. UV (PDA, H_2O/MeCN, nm) 213, 230(sh), 293.

Allyl 2-naphthyl ether (4b2) Allyl bromide and 2-naphthol were reacted. Yellow oilish residue was purified by column chromatography. 89.5% isolation yield. Colorless oil. UV (PDA, H_2O/MeCN, nm) 228.

But-2-enyl 1-naphthyl ethers[5] Crotyl chloride (585μl) was added to the solution of 1-naphthol (0.72g) and K_2CO^3 (1.5 g) in dry DMF (20 mL). The reaction mixture turned to dark black in 10 min. After reacting for 10 hrs, the mixture was filtered, and extracted with diethyl ether (50 mL X 2) after addition of water (50 mL). The collected organic layer was dried with anhydrous Na_2SO_4, and dried under reduced pressure. The orange residue was purified by column chromatography. Colorless oil. ^{1}H-NMR ($CDCl_3$, 600 MHz), δ 1.78 (*m*, 3H, CH_3), 4.63 (*d*, J = 6.0 Hz, 2H, (*E*)-CH_2), 4.78 (*d*, J = 6.0 Hz, 2H, (*Z*)-CH_2), 5.8 – 5.9 (*m*, 2H, vinyl H), 6.81 (*m*, 1H, aromatic H), 7.35 (*m*, 1H, aromatic H), 7.40 (*m*, 1H, aromatic H), 7.47 (*m*, 2H, aromatic H), 7.79 (*m*, 1H, aromatic H), 8.29 (*m*, H, aromatic H). UV (PDA, H_2O/MeCN, nm) 212, 231(sh), 293. Mixture of (*E*)- **(4c1)** and (*Z*)-isomers **(4d1)** (3:1 by ^{1}H-NMR) were separated from HPLC.

---

[5] Tsai, Jui-Chi, et al. "Synthesis of substituted 2, 5-dihydro-1-naphthoxepines from 1-naphthol via ring-closing metathesis." Arkivoc 2008.12 (2008): 205-217.

But-2-enyl 2-naphthyl ethers Crotyl chloride (585μl) and 2-naphthol (0.72g) were reacted. The reaction mixture turned immediately purple. Colorless oil. ^{1}H-NMR ($CDCl_3$, 600 MHz), δ 1.78 (*d*, *J* = 6.0 Hz, 3H, CH_3), 4.57 (*d*, *J* = 6.0 Hz, 2H, (*E*)-CH_2), 4.71 (*d*, *J* = 4.2 Hz, 2H, (*Z*)-CH_2), 5.8 – 5.9 (*m*, 2H, vinyl H), 7.15 (*m*, 2H, aromatic H), 7.32 (*m*, 1H, aromatic H), 7.42 (*m*, 1H, aromatic H), 7.71 (*m*, 3H, aromatic H). Mixture of (*E*)- **(4c2)** and (*Z*)-isomers **(4d2)** (4:1 by ^{1}H-NMR) were separated from HPLC.

Prenyl 1-naphthyl ether (4e1) Prenyl bromide (693μl) and 1-naphthol (0.72g) were reacted. White solid. ^{1}H-NMR ($CDCl_3$, 600 MHz), δ 1.78 (*s*, 3H, CH_3), 1.83 (*s*, 3H, CH_3), 4.70 (*d*, *J* = 6.6 Hz, 2H, CH_2), 5.62 (*m*, 1H, vinyl H), 6.82 (*m*, 1H, aromatic H), 7.35 (*m*, 1H, aromatic H), 7.40 (*m*, 1H, aromatic H), 7.47 (*m*, 2H, aromatic H), 7.79 (*m*, 1H, aromatic H), 8.28 (*m*, H, aromatic H).

Prenyl 2-naphthyl ether (4e2) Prenyl bromide (2.1 g) was added to the solution of 2-naphthol (1.44g) and K_2CO_3 (3 g) in dry DMF (30 mL). The reaction mixture turned to orange immediately, and was reacted for 10 hrs. The product was filtered and extracted with diethyl ether. White solid. ^{1}H-NMR ($CDCl_3$, 600 MHz), δ 1.79 (*s*, 3H, CH_3), 1.82 (*s*, 3H, CH_3), 4.63 (*d*, *J* = 7.2 Hz, 2H, CH_2), 5.56 (*m*, 1H, vinyl H), 7.16 (*m*, 2H, aromatic H), 7.32 (*m*, 1H, aromatic H), 7.42 (*m*, 1H, aromatic H), 7.72 (*m*, 3H, aromatic H).

(2-Methylallyl) 1-naphthyl ether (4f1)[6] Under N_2 atmosphere, 3-bromo-2-methylpropene (1.0 mL) was added to the solution of 1-naphthol (726 mg) in dry acetone. The reaction was initiated by addition of K_2CO_3 (1.74 g). The reaction mixture was stirred for 10 hrs at room

6 Boddy, I. K., Cambie, R. C., Dixon, G., Rutledge, P. S., & Woodgate, P. D. (1983). Experiments directed towards the synthesis of anthracyclinones. VII. Model studies with allyl naphthalenyl ethers. Australian Journal of Chemistry, 36(4), 803-813.

temperature. The reaction mixture was filtered and the filtrate was worked-up, and the product was purified by column chromatography. yellow oil. ^{1}H-NMR ($CDCl_3$, 600 MHz), δ 1.92 (*s*, 3H, CH_3), 4.61 (*s*, 2H, CH_2), 5.04 (*s*, 1H, vinyl H), 5.21 (*s*, 1H, vinyl H), 6.80 (*m*, 1H, aromatic H), 7.35 (*m*, 1H, aromatic H), 7.41 (*m*, 1H, aromatic H), 7.48 (*m*, 2H, aromatic H), 7.79 (*m*, 1H, aromatic H), 8.31 (*m*, H, aromatic H). UV (PDA, H_2O/MeCN, nm) 211, 231(sh)

(2-Methylallyl) 2-naphthyl ether (4f2) white crystalline solid. ^{1}H-NMR ($CDCl_3$, 600 MHz), δ 1.88 (*s*, 3H, CH_3), 4.56 (*s*, 2H, CH_2), 5.03 (*s*, 1H, vinyl H), 5.16 (*s*, 1H, vinyl H), 7.15 (*m*, 1H, aromatic H), 7.17 (*m*, 1H, aromatic H), 7.33 (*m*, 1H, aromatic H), 7.43 (*m*, 1H, aromatic H), 7.75 (*m*, 3H, aromatic H). UV (PDA, H_2O/MeCN, nm) 227

Propargyl 2-naphtyl ether (4g2) Under N_2 atmosphere, propargyl bromide (1.54 g) was added to the solution of 2-naphthol (1.41 g) and K_2CO_3 (3 g) in dry DMF. The reaction mixture was refluxed for 10 hrs and the product was purified by silica gel column chromatography. ^{1}H-NMR ($CDCl_3$, 600 MHz), δ 2.55 (*s*, H, ≡CH), 4.81 (*d*, *J* = 2.4 Hz, 2H, CH_2), 7.20 (*m*, 1H, aromatic H), 7.24 (*m*, 1H, aromatic H), 7.36 (*m*, 1H, aromatic H), 7.45 (*m*, 1H, aromatic H), 7.76 (*m*, 3H, aromatic H). UV (PDA, H_2O/MeCN, nm) 227

2-(naphthalen-1-yloxy)acetonitrile (4h1) Bromoacetonitrile (420 μL, 5.02 mmol) was added to the mixture of 1-naphthol (712 mg, 4.89 mmol) and K_2CO_3 (2.09 g) in dry acetone (20 mL) and the reaction mixture was stirred for 24 hours at room temperature. The reaction mixture was purred into distilled water (150 mL) and the organic fraction was extracted by diethyl ether (150 mL) twice. The combined organic fraction was evaporated and the product was purified by silica gel column chromatography. Rf = 0.66 (hexanes:ethyl acetate = 5:2) ^{1}H NMR ($CDCl_3$, 600 MHz), δ 4.92 (*s*, 2H, CH_2), 6.89 (*m*, 1H, aromatic H), 7.39 (*m*, 1H, aromatic H), 7.52 (*m*, 3H, aromatic H), 7.82 (*m*, 1H, aromatic H), 8.19 (*m*, 1H, aromatic H) ^{13}C NMR ($CDCl_3$, 150

MHz), δ 53.7 (CH_2), 105.1, 115.1 (C≡N), 121.5, 122.8, 125.3x2, 126.0, 127.0, 127.7, 134.7, 152.4.

2-(naphthalen-2-yloxy)acetonitrile (4h2) Bromoacetonitrile (418 μL, 5.00 mmol) was added to the mixture of 2-naphthol (722 mg, 4.96 mmol) and K_2CO_3 (2.13 g) in dry acetone and the reaction mixture was stirred for 12 hrs at room temperature. The reaction mixture was purred into distilled water (150 mL) and the organic fraction was extracted by diethyl ether (mL) twice. The combined organic fraction was evaporated and the product was purified by silica gel column chromatography. Rf = 0.68 (hexanes:ethyl acetate = 5:2) ^{1}H NMR ($CDCl_3$, 600 MHz), δ 4.89 (*s*, 2H, CH_2), 7.19 (*m*, 1H, aromatic H), 7.25 (*m*, 1H, aromatic H), 7.41 (*m*, 1H, aromatic H), 7.50 (*m*, 1H, aromatic H), 7.80 (*m*, 3H, aromatic H) ^{13}C NMR ($CDCl_3$, 150 MHz), δ 53.5 (CH_2), 107.8, 115.0 (C≡N), 118.1, 124.8, 126.9, 127.1, 127.7, 129.9,130.2, 134.0, 154.5.

S2. Biotransformation of veratrole (2) and 4,5-dibromovertrole (3)

The study of 4,5-dibromoveratrole metabolism by MRG-PMF1. PMF1 was inoculated in GAM broth medium (2 mL) in the anaerobic chamber comprising 5% CO_2, 10% H_2, and 85% N_2 at 35°C. Veratrole and 4,5-dibromoveratrole were dissolved in DMF (concentration 10 mM). The solution of veratrole and 4,5-dibromoveratrole (40 µL) were reacted with 4 mL of cell cultures (OD_{600} = 0.9). After 0 h, 3, 6, 9, 24 h, 3 d, 4 d and 5 d incubation, 100 µL of reaction mixture was extracted with 1 mL of ethyl acetate which was vortexed for 20s and centrifuged for 10 min (10770 g). Then 800 µL of supernatant was taken and dried under reduced pressure. The residue was dissolved in 100 µL of DMF and filtered through 0.2 µm filter (Grace, MD, USA) for HPLC analysis.

The reaction products were analyzed via Finnigan Surveyor Plus HPLC with Thermo PDA Plus detector, equipped with a C18 Hypersil GOLD™ column (4.6×100 nm; 5 µm; Thermo Scientific, Waltham, MA, USA) was used for HPLC analyses. The injection volume was 10 µL and the flow was 1.0 mL/min. For the HPLC analysis of veratrole and 4,5-dibromoveratrole metabolites, the mobile phase was comprised of 0.1% acetic acid in deionized water (solvent A) and 0.1% acetic acid in acetonitrile (solvent B). The solution B was start at 20% and increased to 40% in 8 min, to 50% in 12 min, to 55% in 15 min, to 70% in 20 min, to 80% in 22 min, and finally to 20% in 30 min.

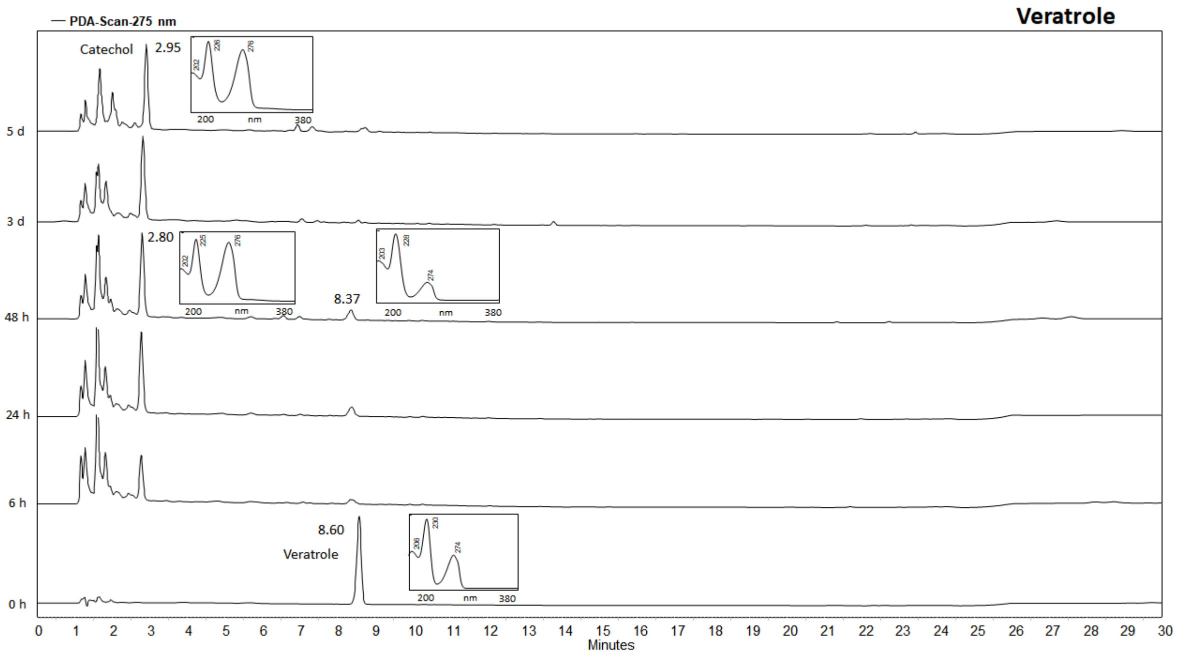
PDA-Scan-275 nm
Veratrole
Catechol
2.95
2.80
8.37
8.60
Veratrole
5 d
3 d
48 h
24 h
6 h
0 h
Minutes

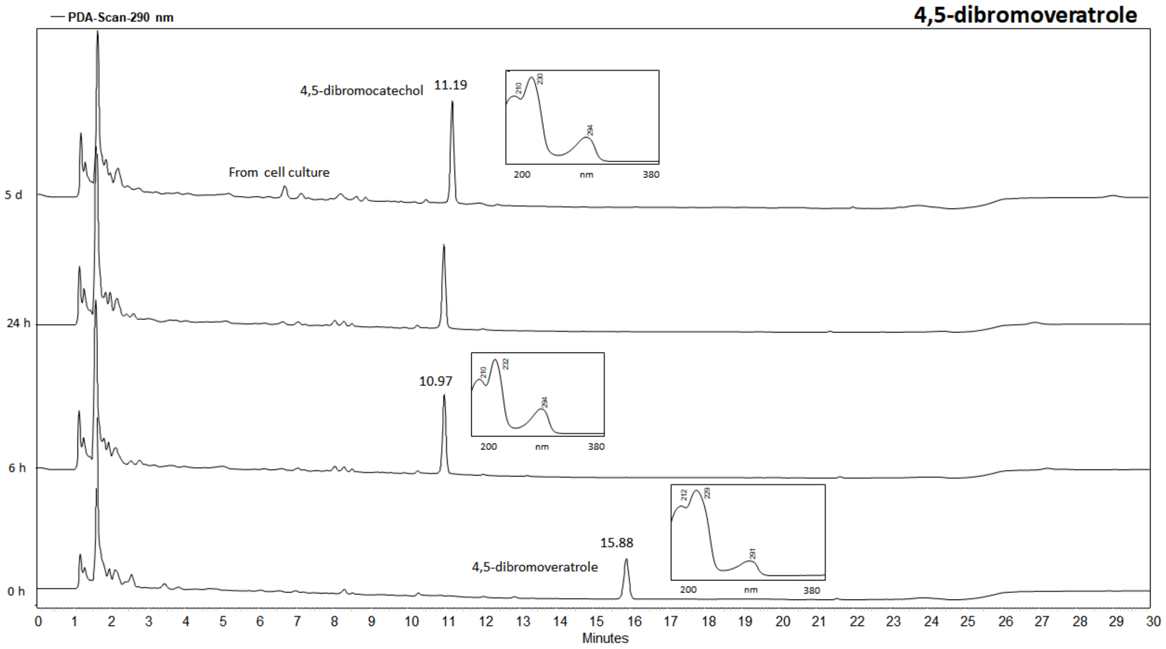
PDA-Scan-290 nm
4,5-dibromoveratrole
4,5-dibromocatechol
11.19
From cell culture
10.97
15.88
4,5-dibromoveratrole
5 d
24 h
6 h
0 h
Minutes

S3. HPLC analysis of 4a biotransformation

Biotransformation of methyl naphthyl ethers was monitored by HPLC. Conversion of **4a1** and **4a2** was monitored at the wavelength of 231nm and 225nm, respectively.

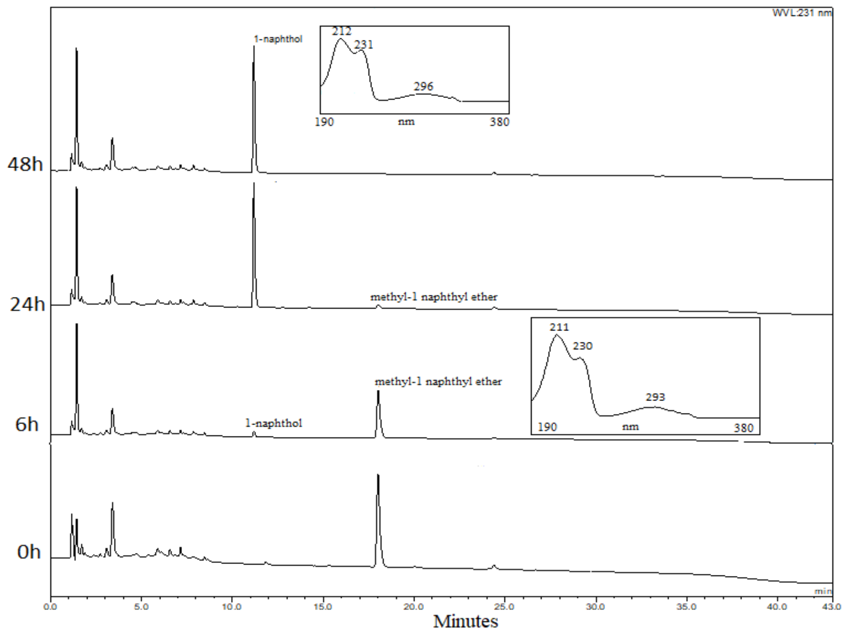

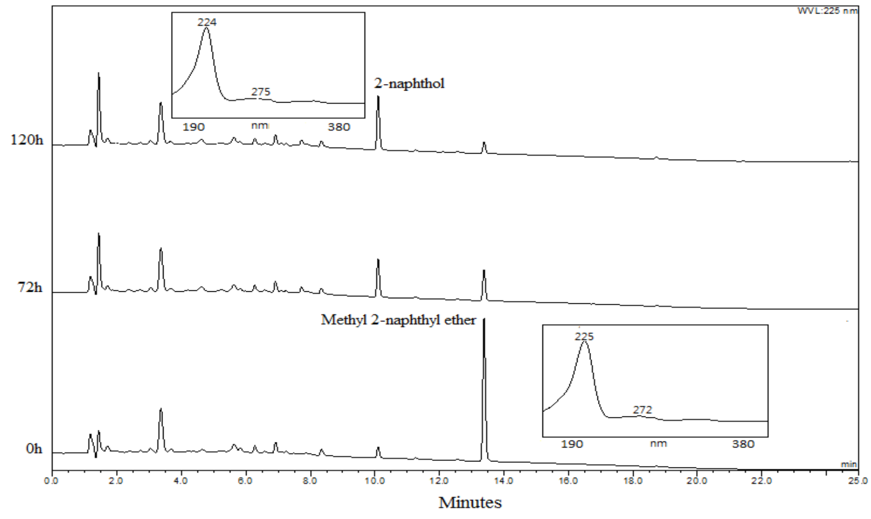

S4. HPLC analysis of 4c2 and 4d2 biotransformation

Biotransformation of a mixture of *trans*-but-2-enyl 2-naphthyl ether (**4c2**) and *cis*-but-2-enyl 2-naphthyl ether (**4d2**) allyl naphthyl ethers was monitored by HPLC at the wavelength of 231 nm.

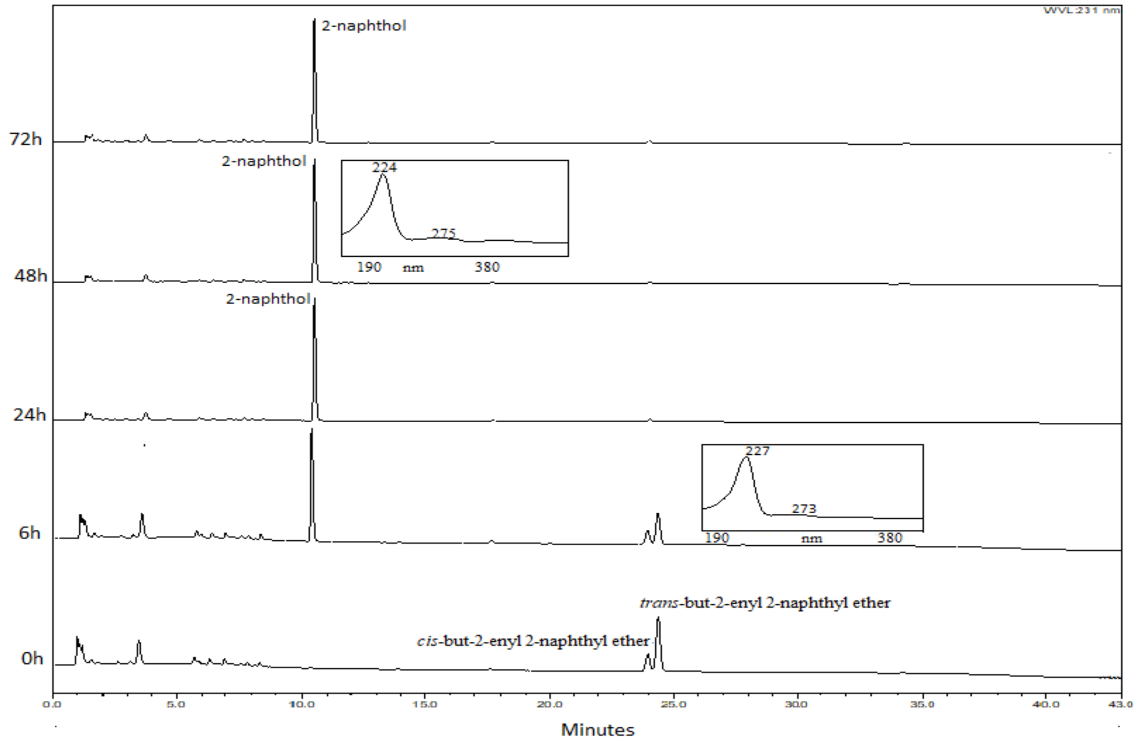

S5. HPLC analysis of a mixture of but-2-enyl 2-naphthyl ether (4c and 4d) biotransformation

Conversion of a mixture of four isomers was monitored by HPLC at the wavelength of 231 nm.

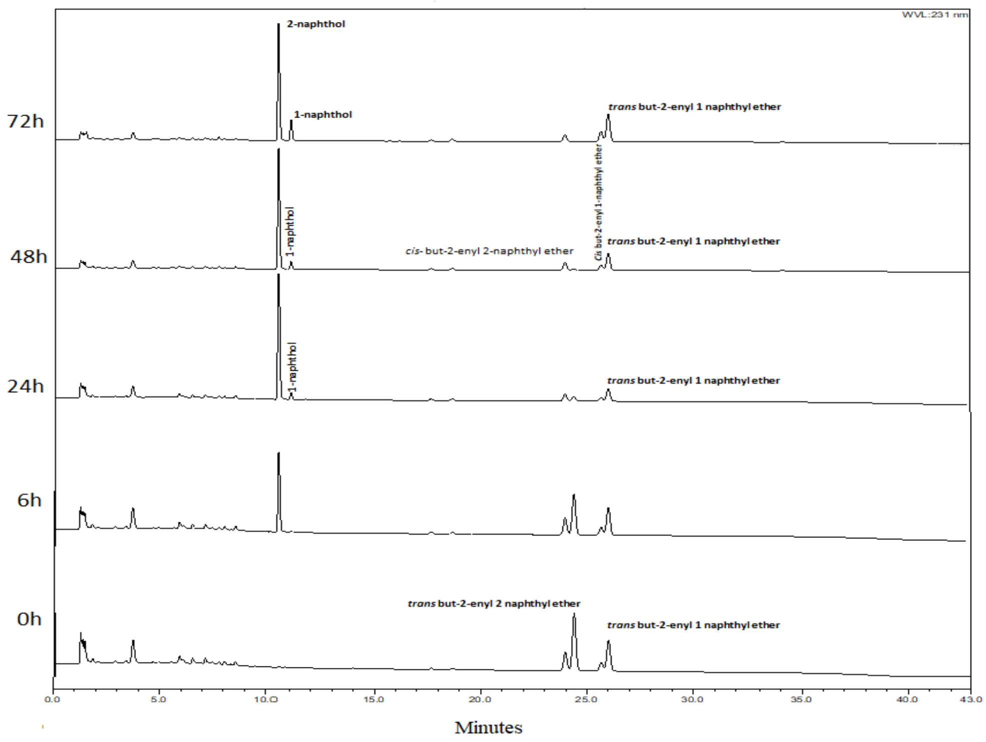

S6. HPLC analysis of 4e biotransformation

Conversions of **4e1** and **4e2** were monitored at the wavelength of 231nm.

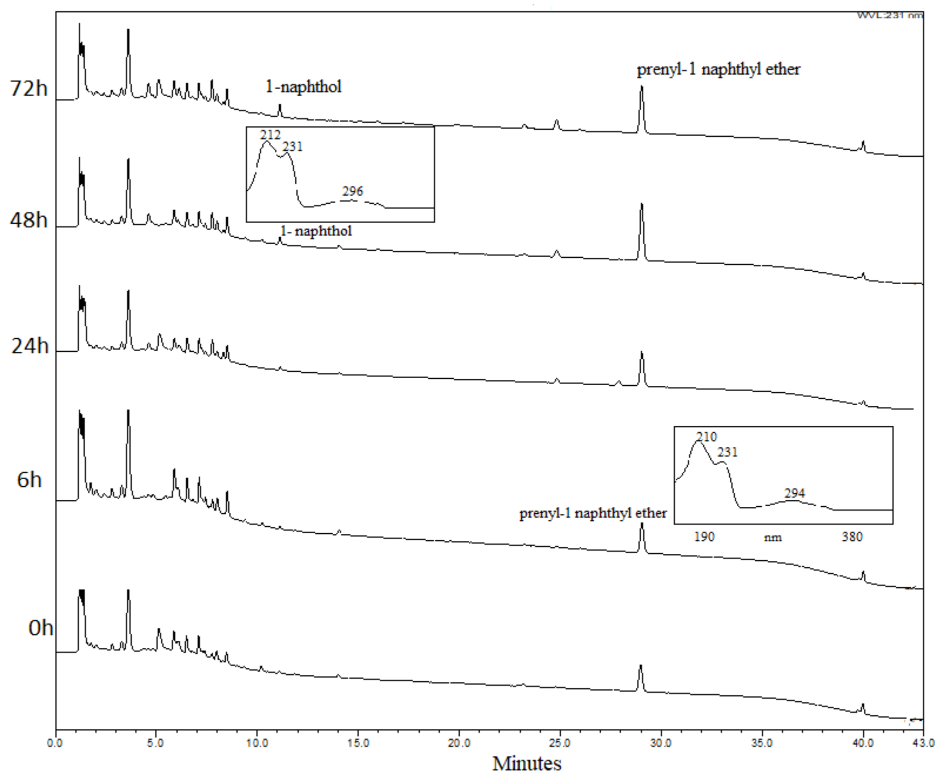

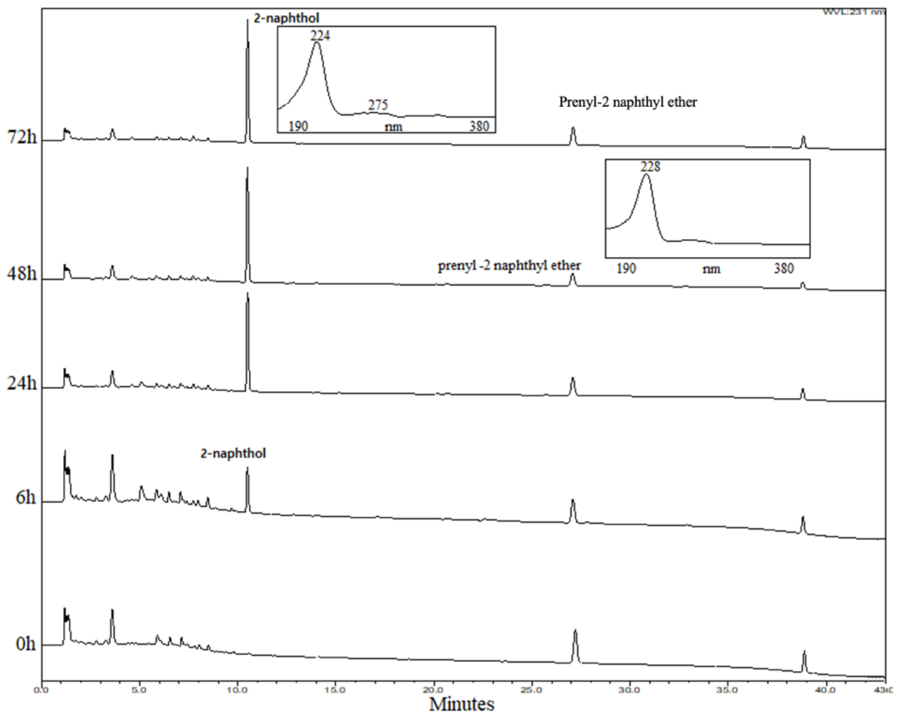

S7. HPLC analysis of 4f biotransformation

Conversion of (2-methylallyl) naphthyl ethers (**4f**) was monitored by HPLC. Conversions of **4f1** and **4f2** were monitored at the wavelength of 231nm and 225nm, respectively.

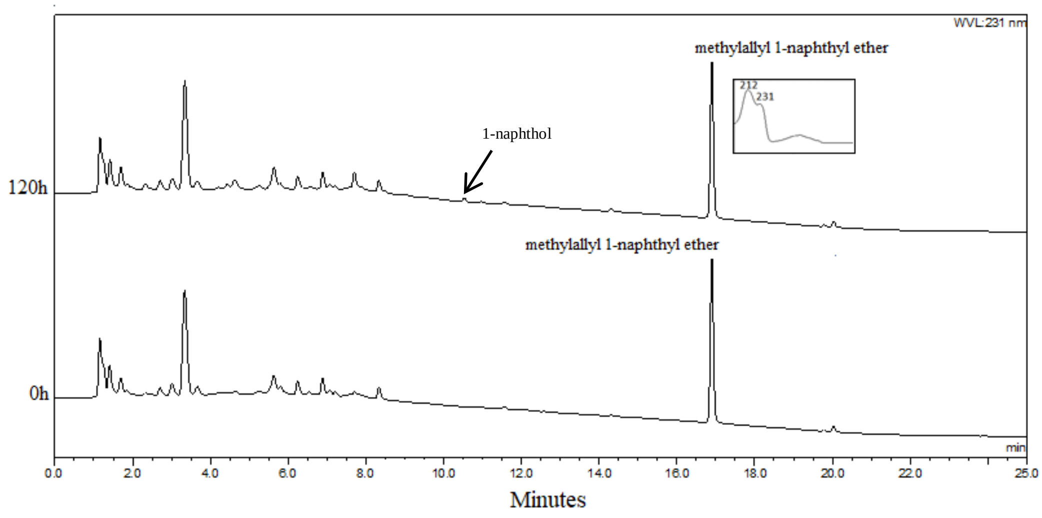

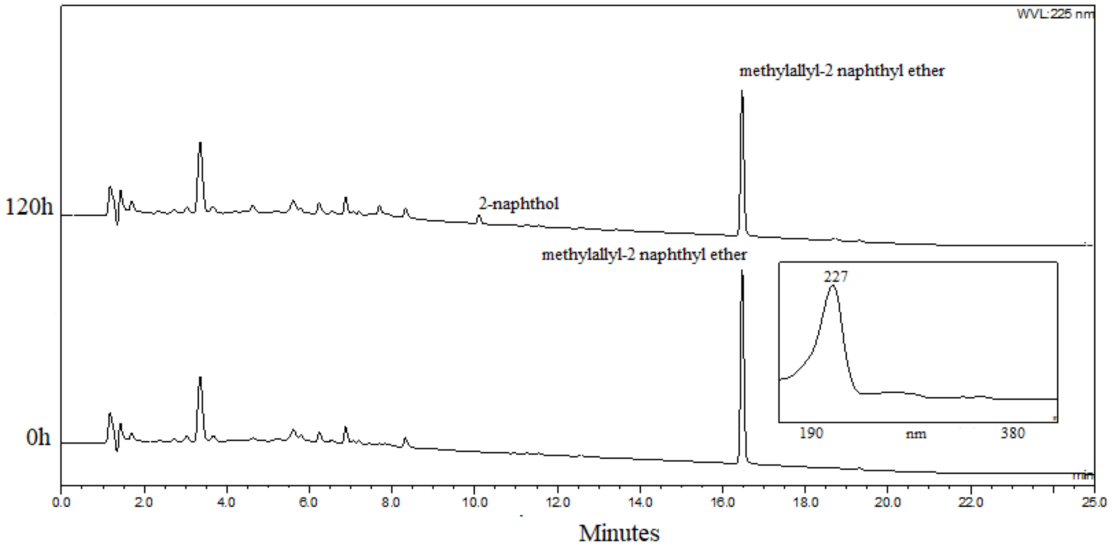

S8. HPLC analysis of 4g biotransformation

The conversion of **4g2** was monitored at 231 nm and no reaction was observed.

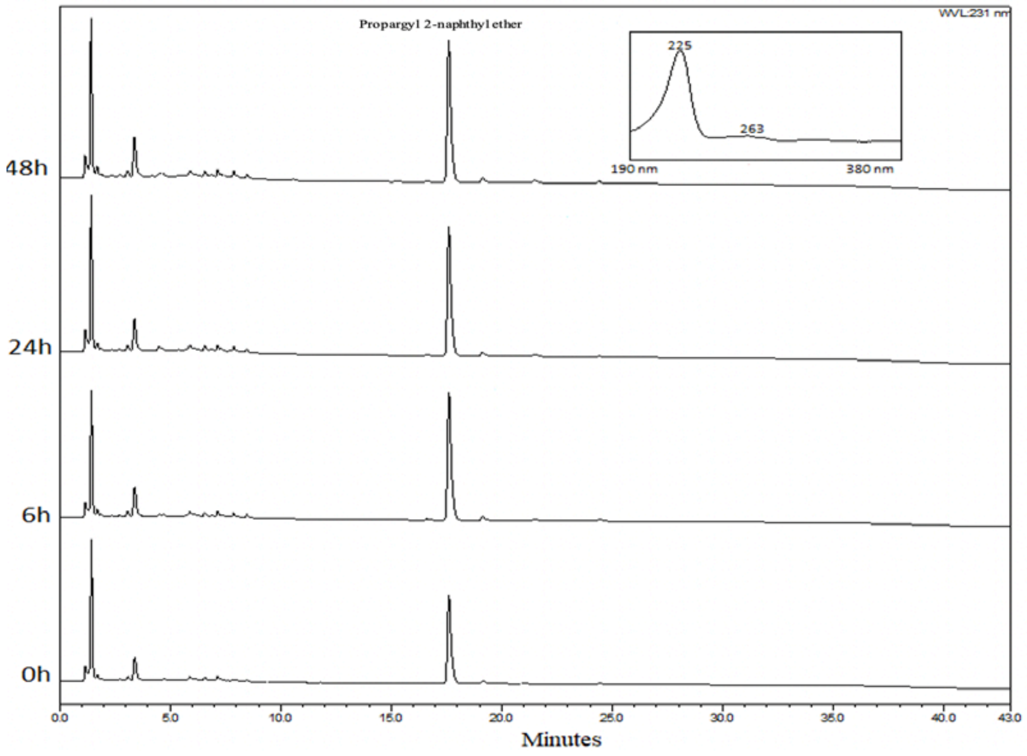

S9. HPLC analysis of 4h biotransformation

The conversion of **4h1** and **4h2** was monitored at 231 nm and no reaction was observed

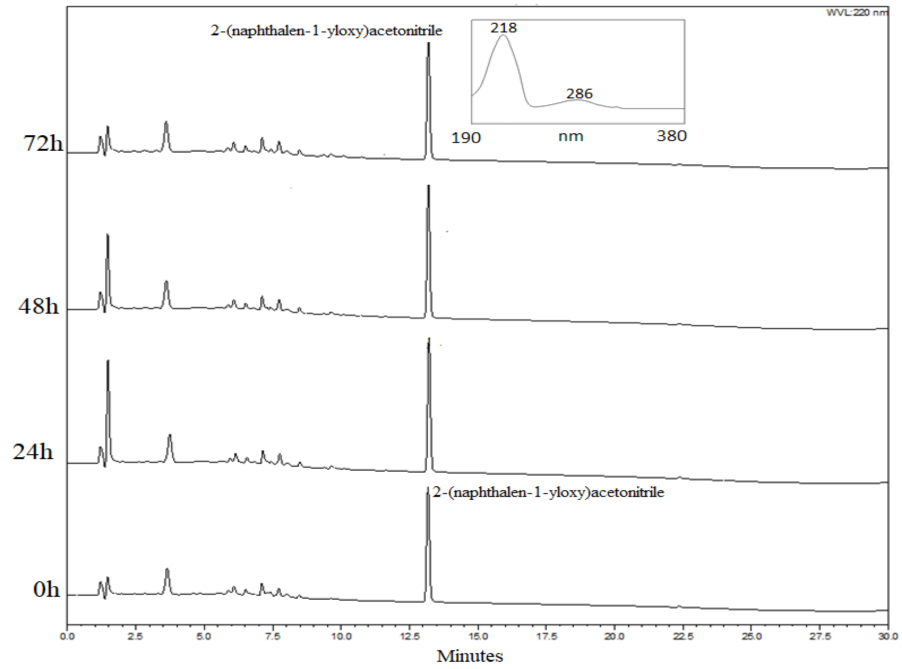

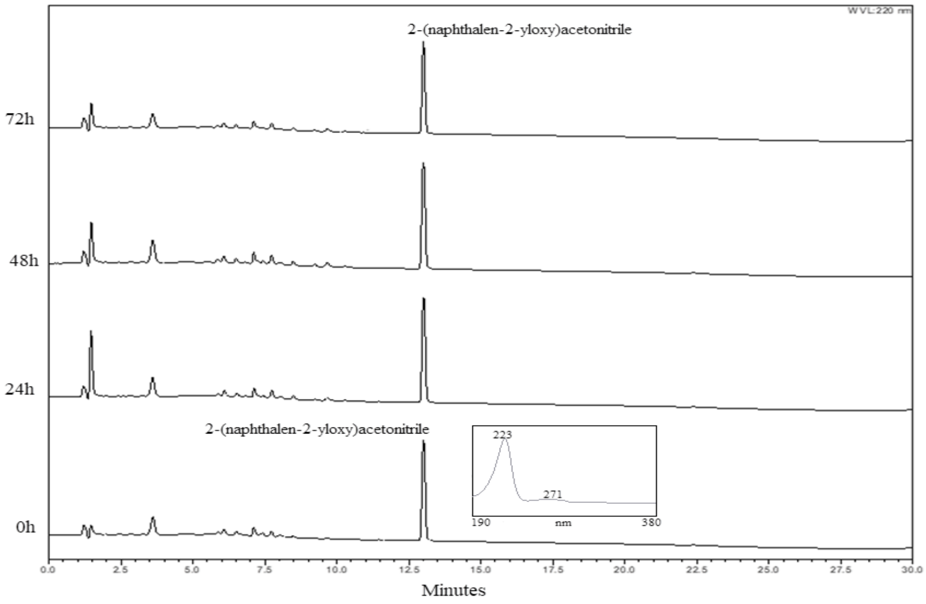

S10. NMR spectra of the compounds

Figure S1. ^{1}H (up) and ^{13}C NMR (down) spectra of the purified isomeric mixture of 4c1 and 4d1

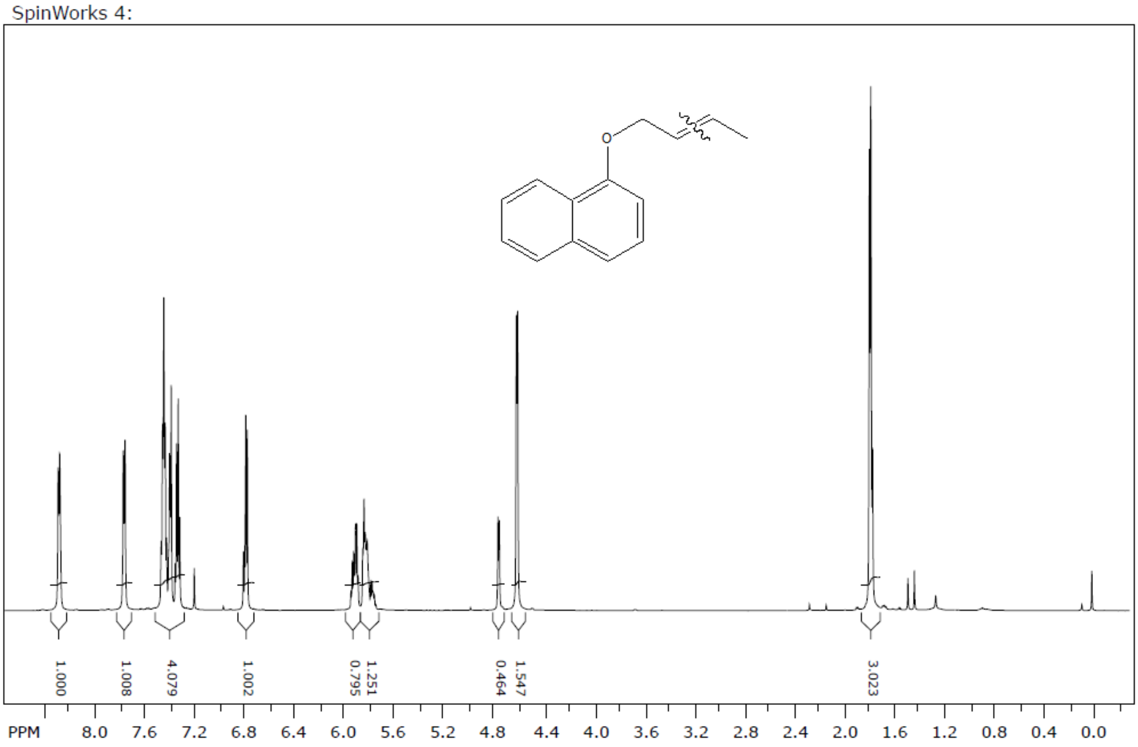

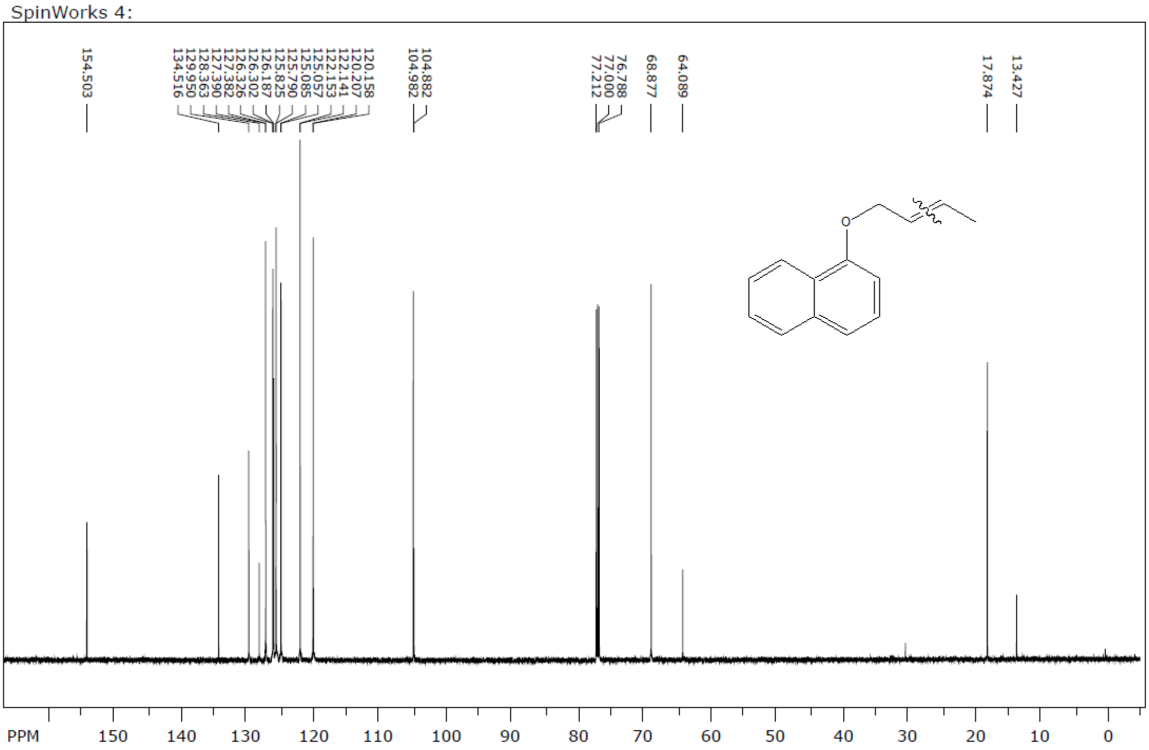

Figure S2. ^{1}**H (up) and** ^{13}**C NMR (down) spectra of the purified isomeric mixture of 4c2 and 4d2**

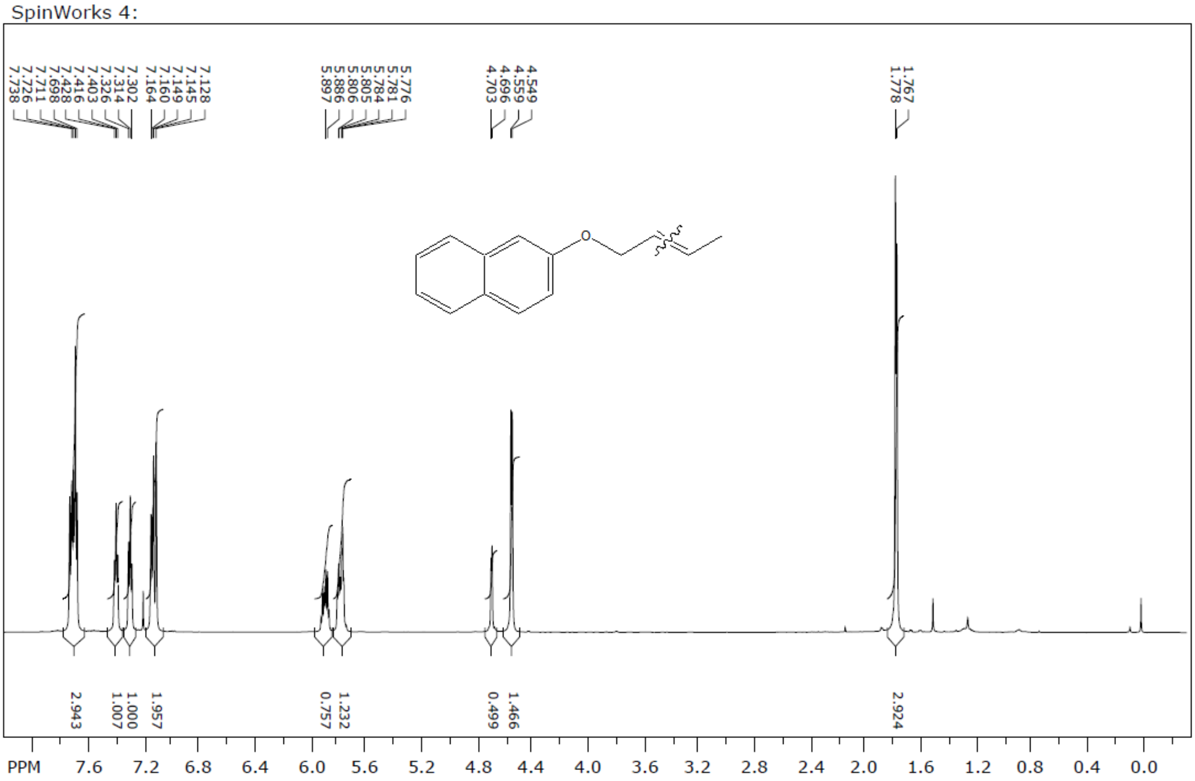

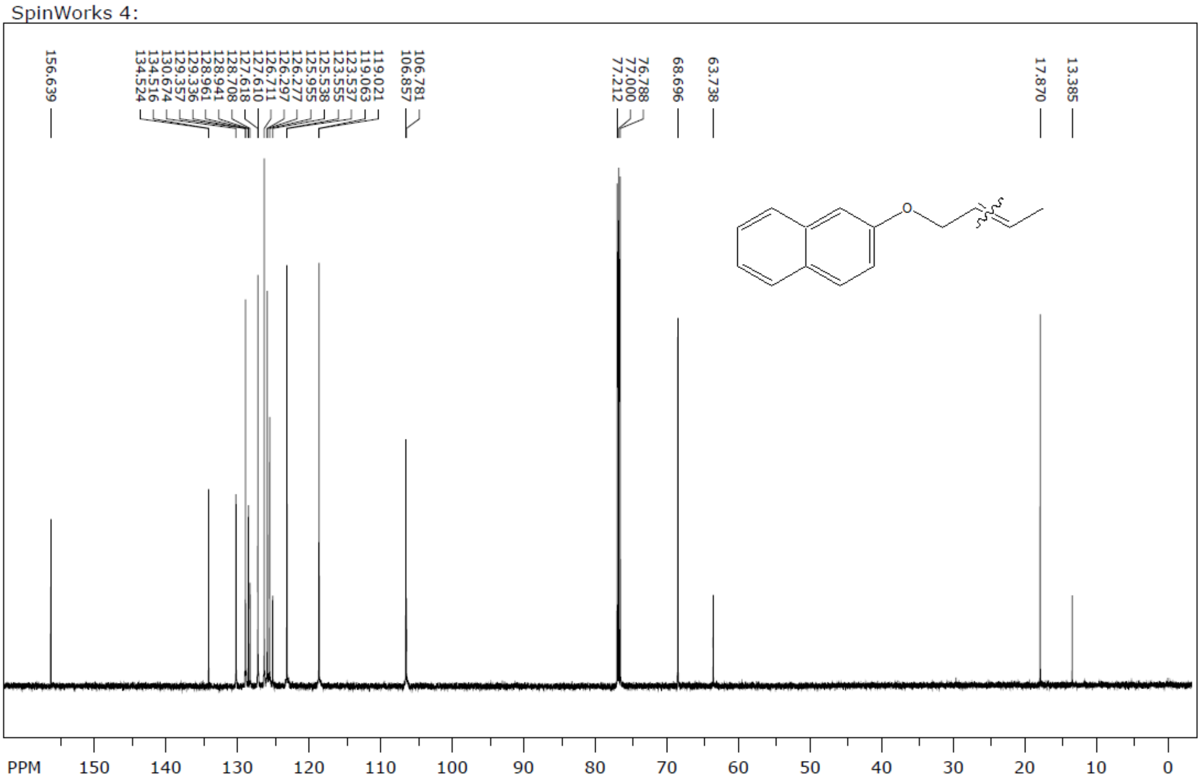

Figure S3. ^{1}H (up) and ^{13}C NMR (down) spectra of 4e1

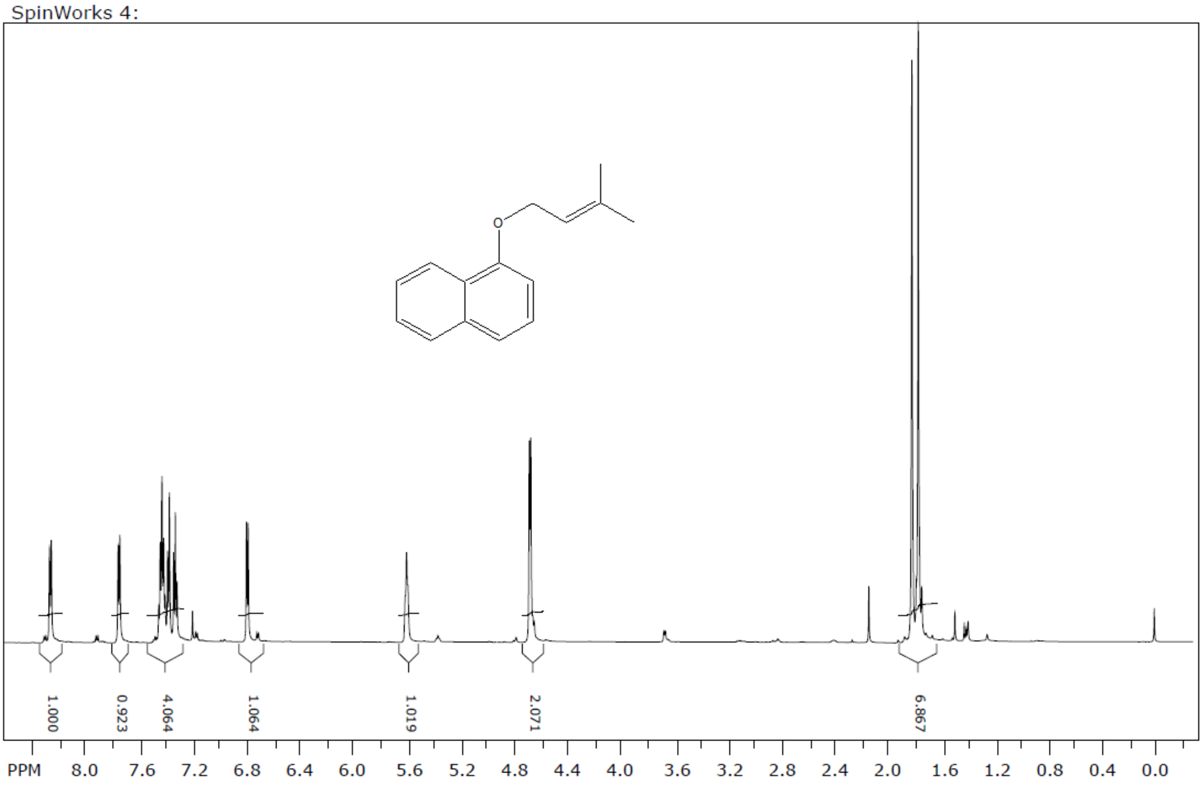

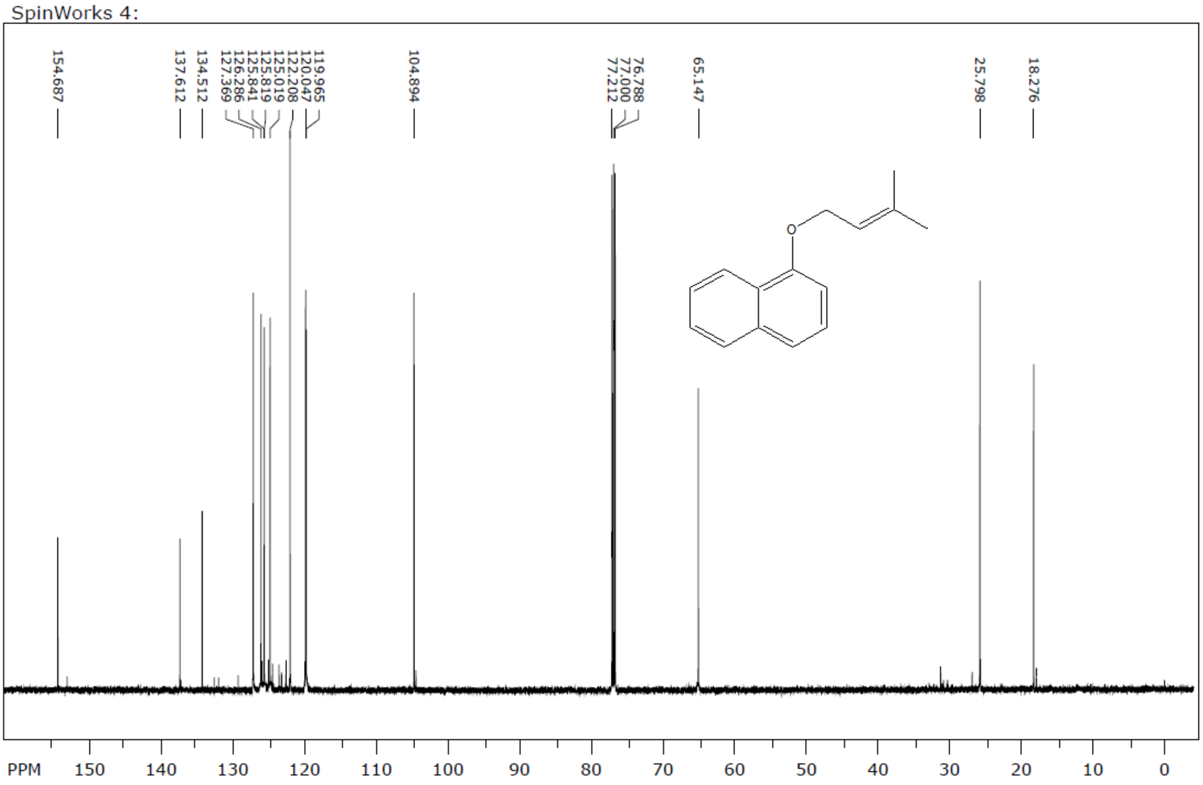

Figure S4. ^{1}H (up) and ^{13}C NMR (down) spectra of 4e2

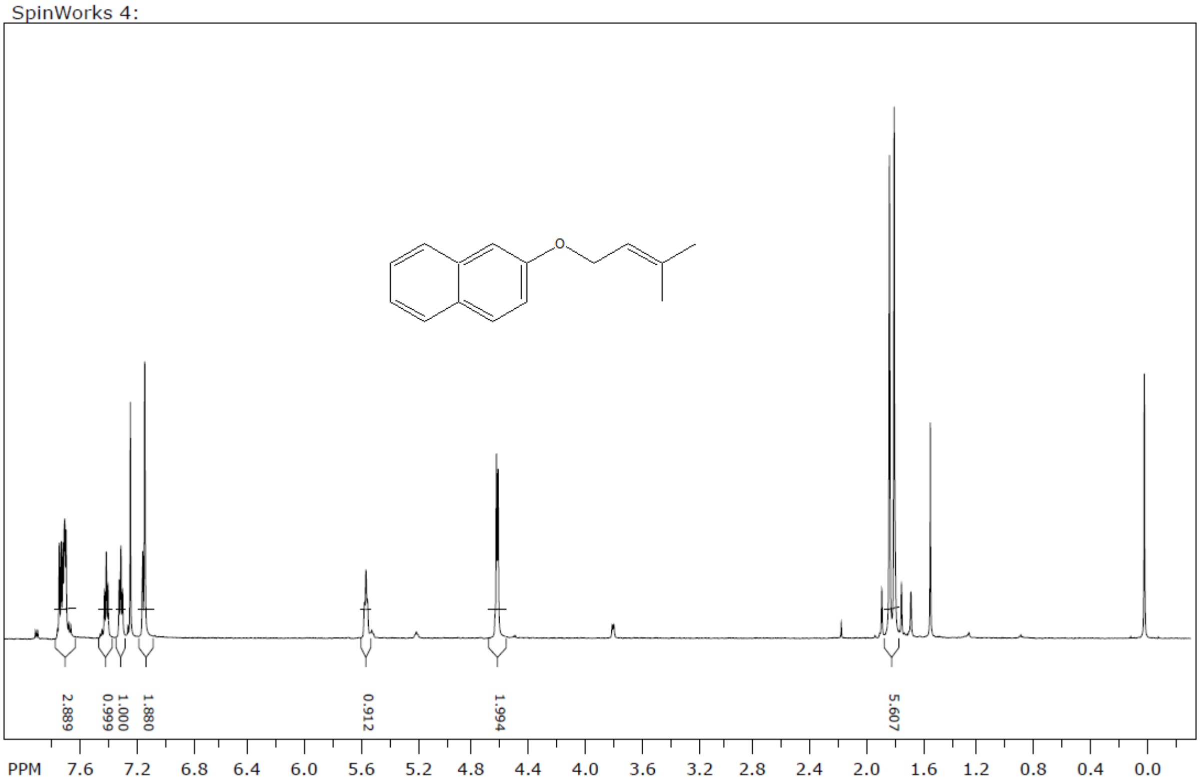

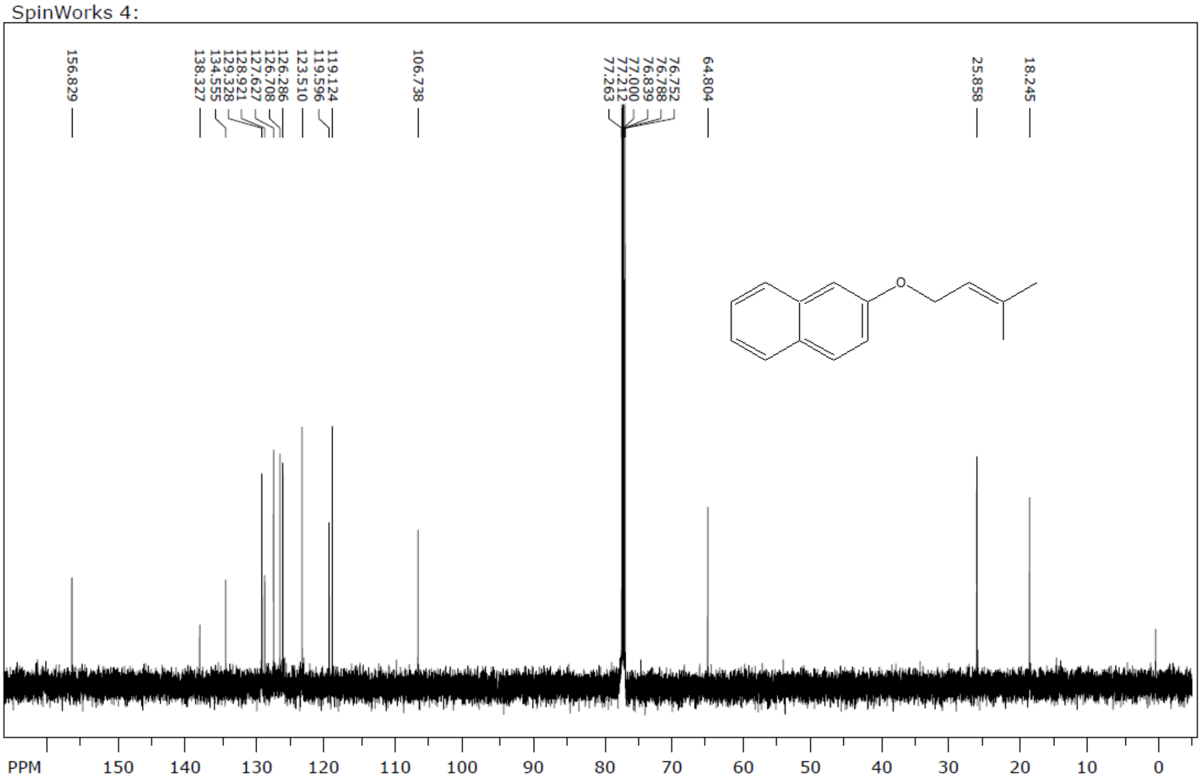

Figure S5. ^{1}H NMR spectrum of 4f1

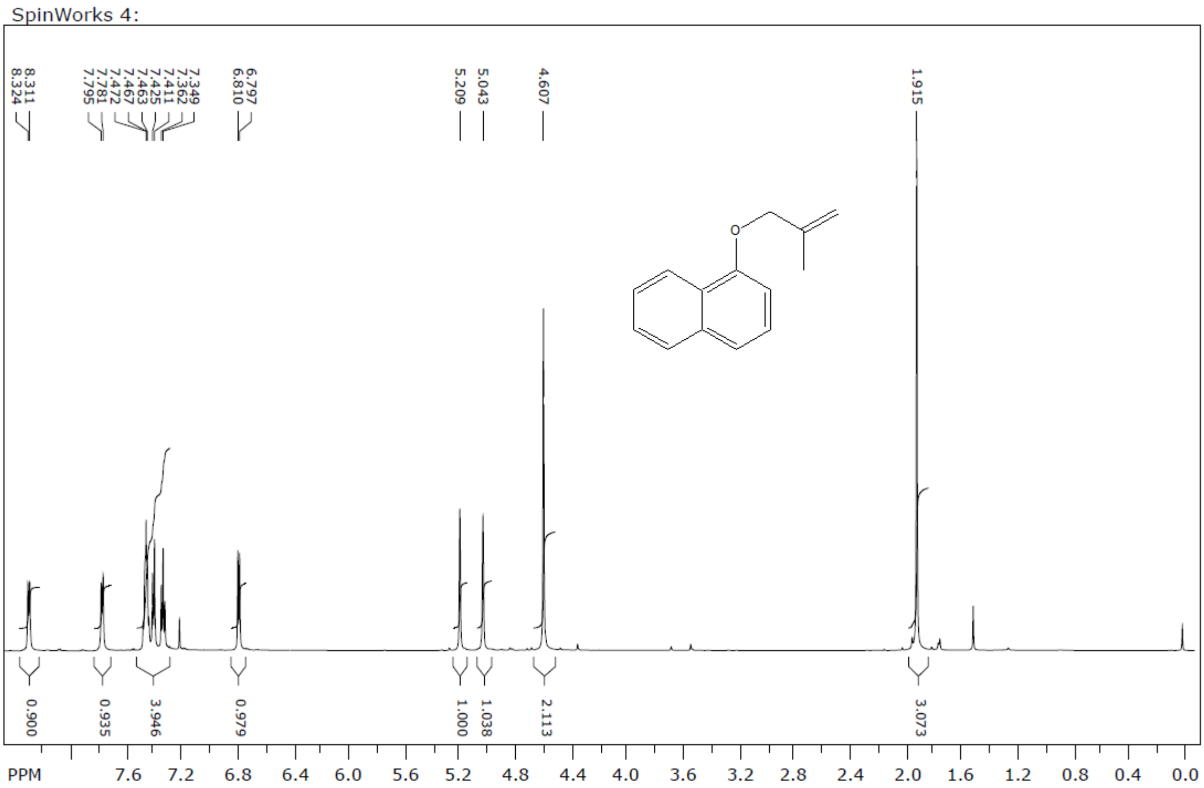

Figure S6. ^{1}H NMR spectrum of 4f2.

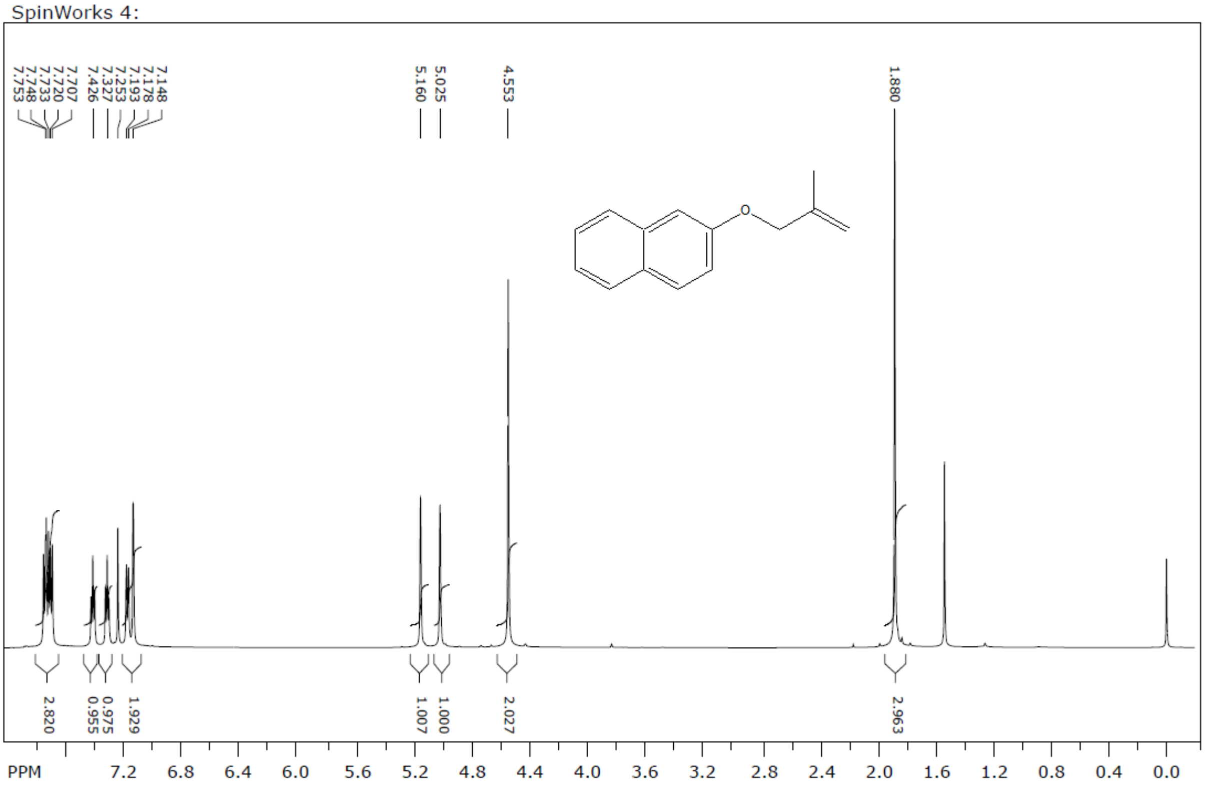

Figure S7. ^{1}H NMR spectra of 4g2

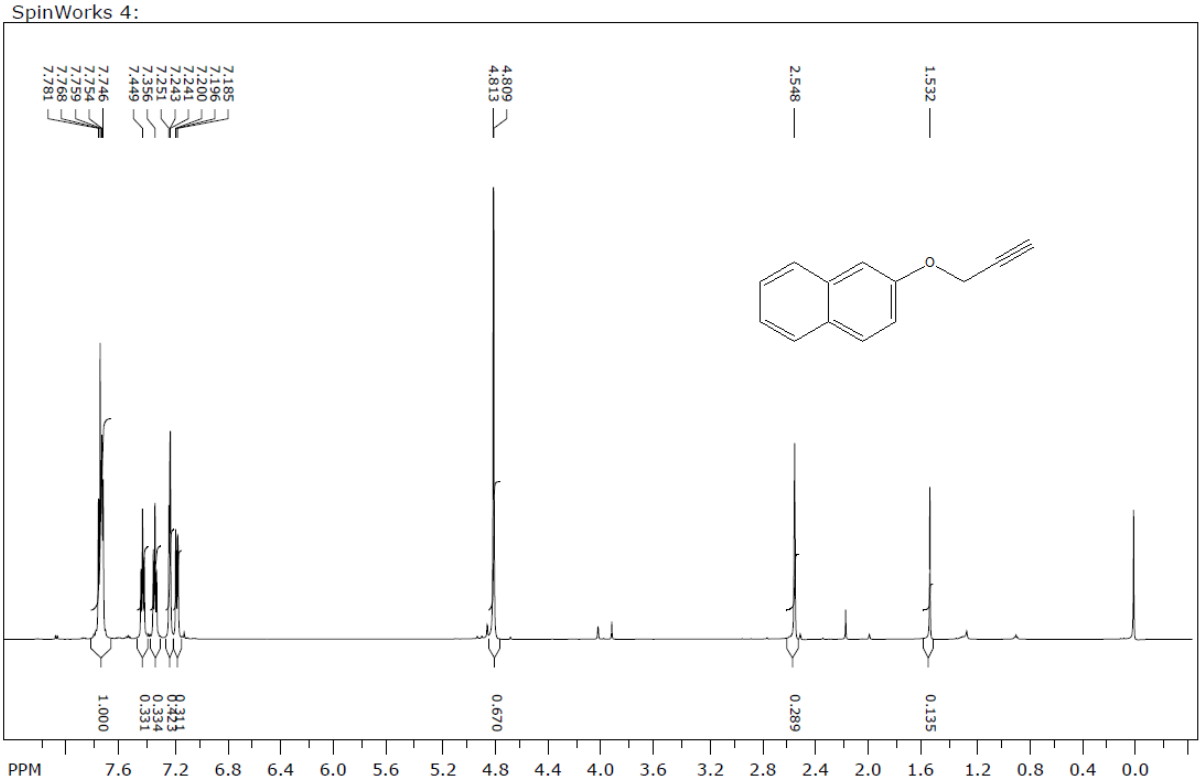

Figure S8. ^{1}H (up) and ^{13}C NMR (down) spectra of 4h1

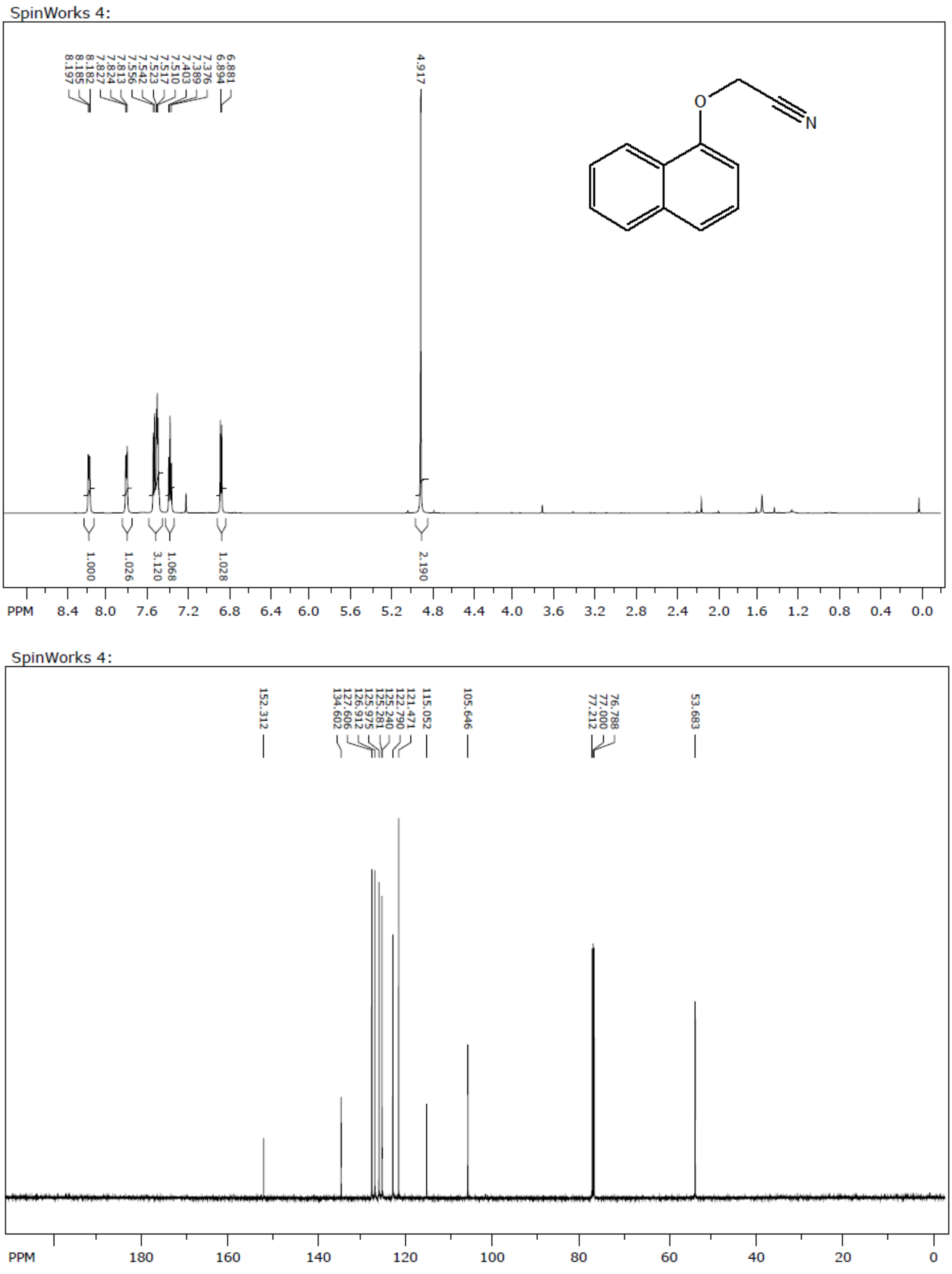

Figure S9. ^{1}H **(up) and** ^{13}C **NMR (down) spectra of 4h2**

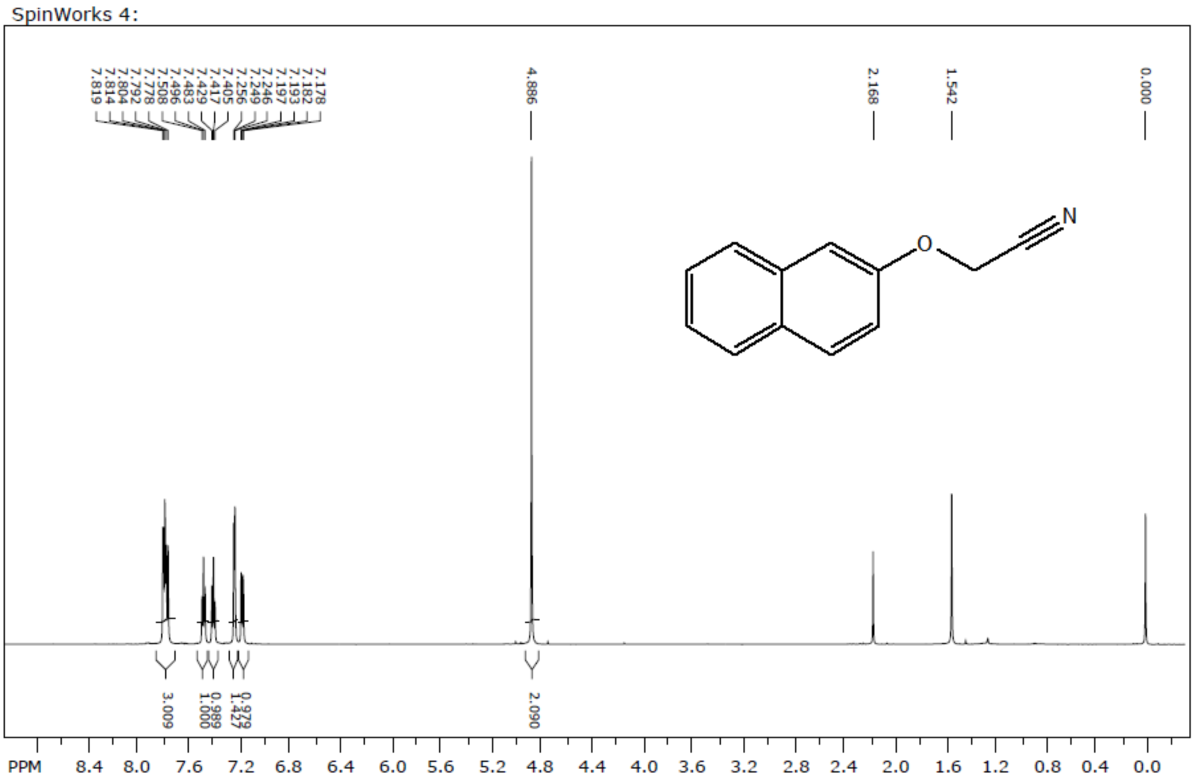

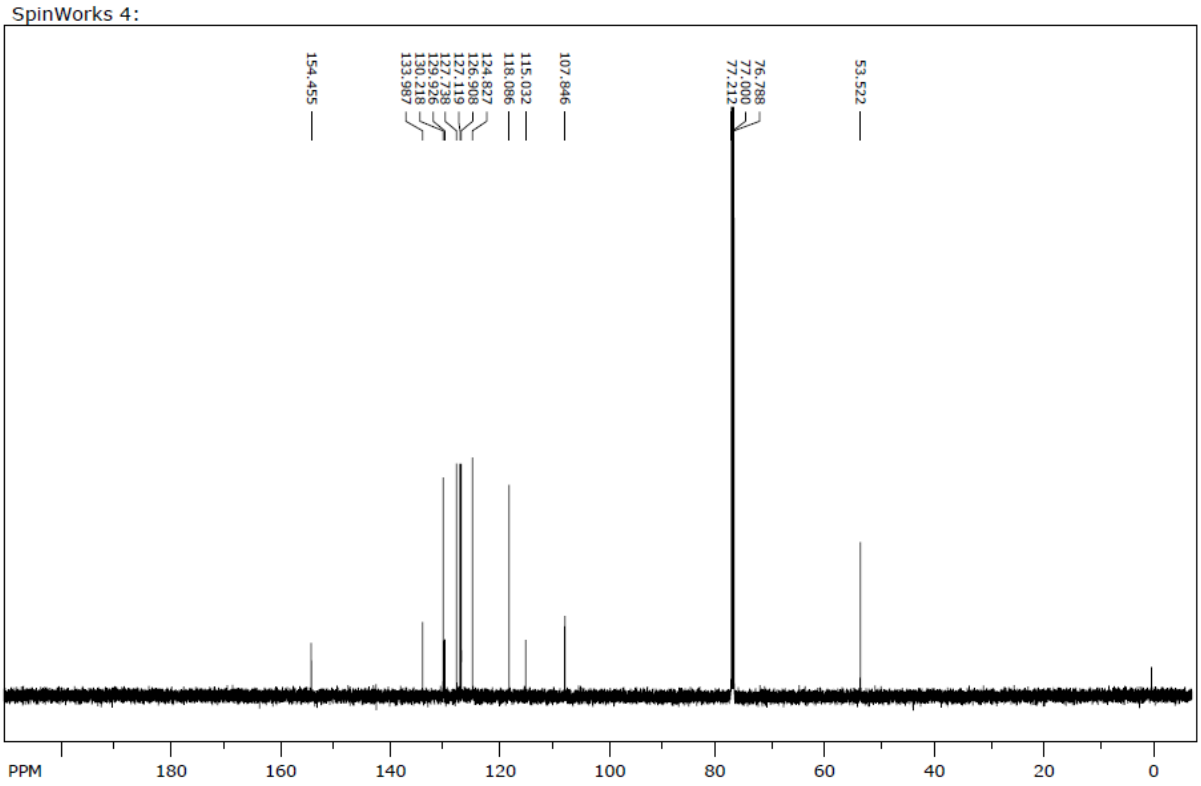

S11. Computational study results

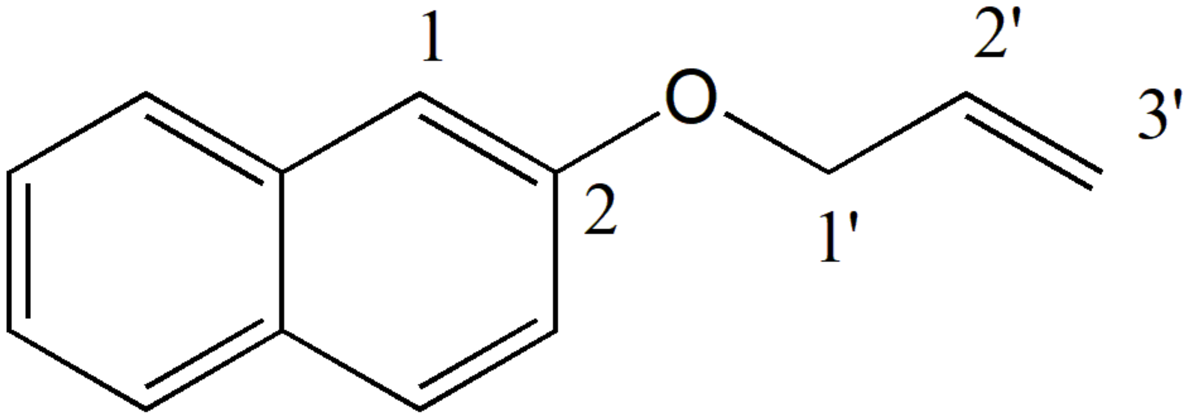

Torsional angle numbers for allyl aryl ether system

A. Allyl 1-naphthyl ether substrates

Geometry-optimized structure (left) and potential energy changes of **4b1**, depending on the torsional angles of C1-O-C1’-C2’ (middle) and O-C1’-C2’-C3’ (right).

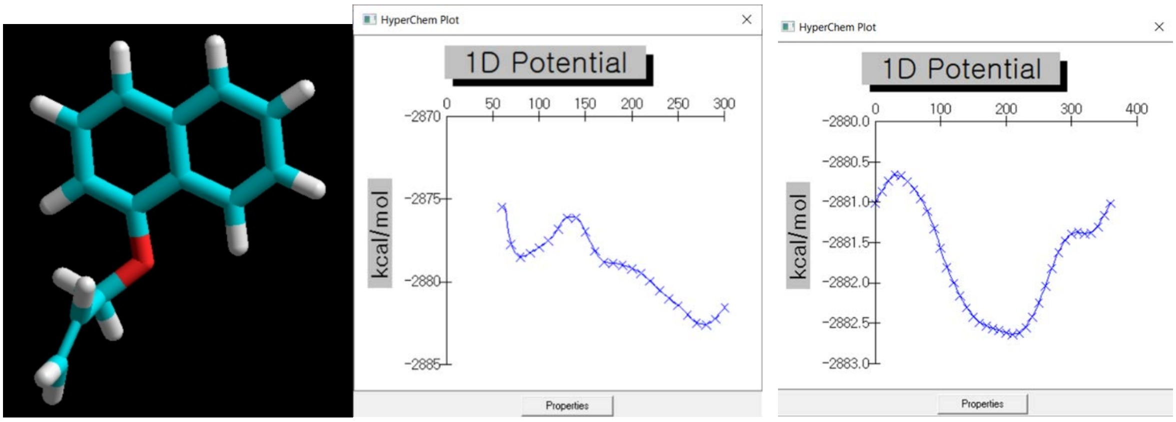

Geometry-optimized structure (left) and potential energy changes of **4c1**, depending on the C1-O-C1’-C2’ (middle) and the O-C1’-C2’-C3’ (right) torsional angle changes

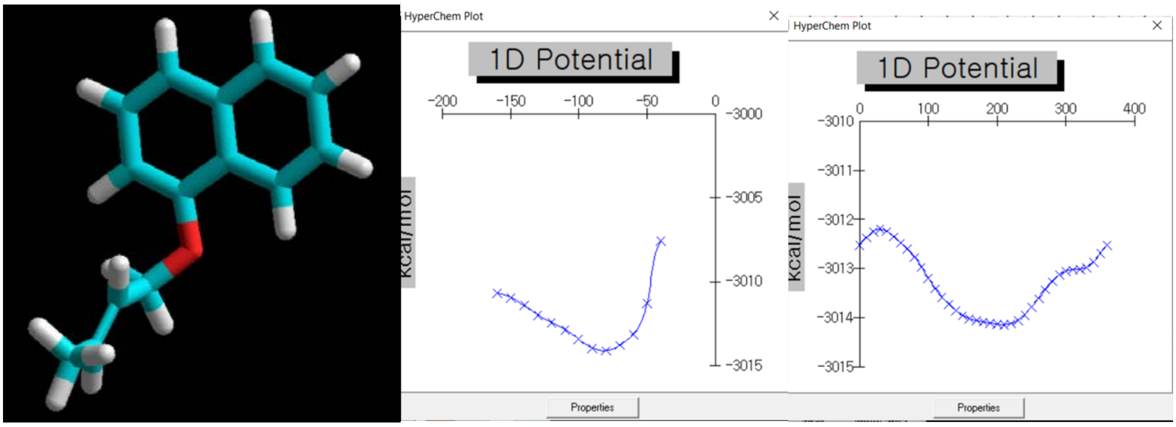

Geometry-optimized structure (left) and potential energy changes of **4d1**, depending on the C1-O-C1’-C2’ (middle) and the O-C1’-C2’-C3’ (right) torsional angle changes

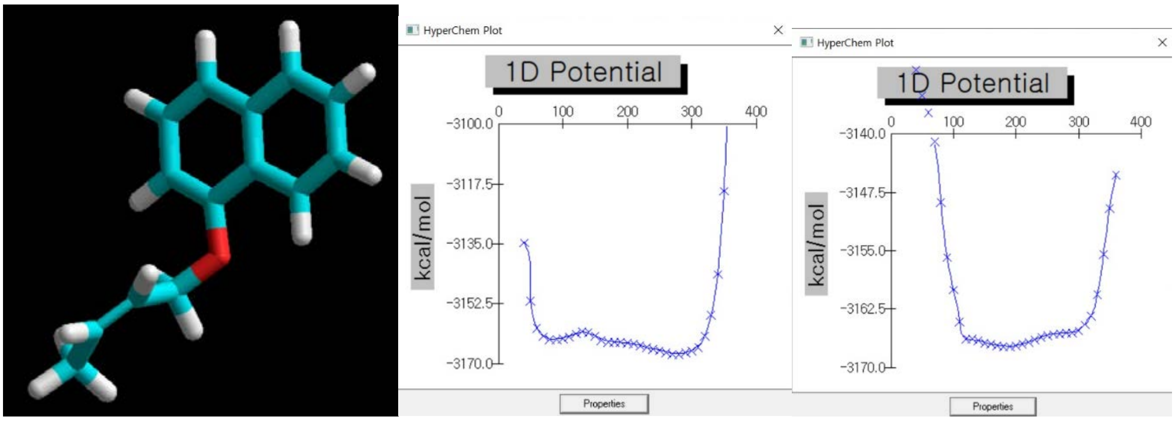

Geometry-optimized structure (left) and potential energy changes of **4e1**, depending on the C1-O-C1’-C2’ (middle) and the O-C1’-C2’-C3’ (right) torsional angle changes

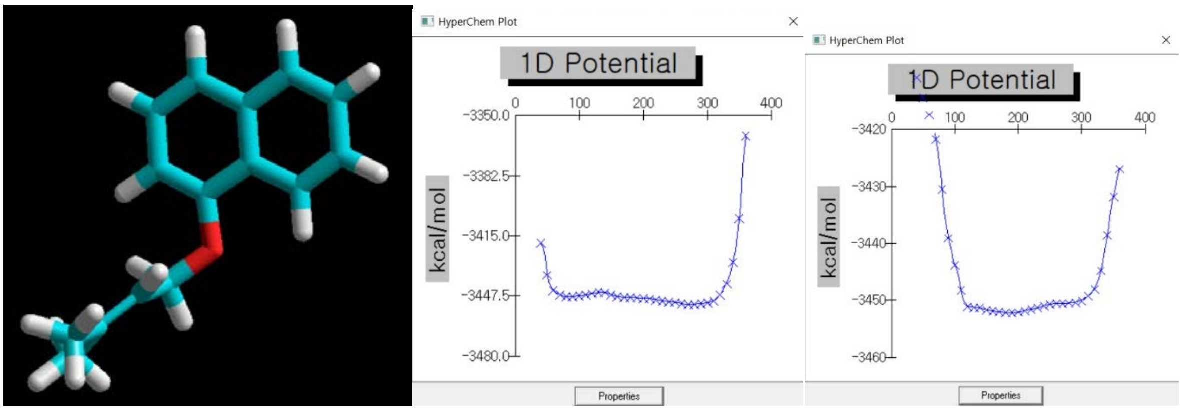

Geometry-optimized structure (left) and potential energy changes of **4f1**, depending on the C1-O-C1'-C2' (middle) and the O-C1'-C2'-C3' (right) torsional angle changes

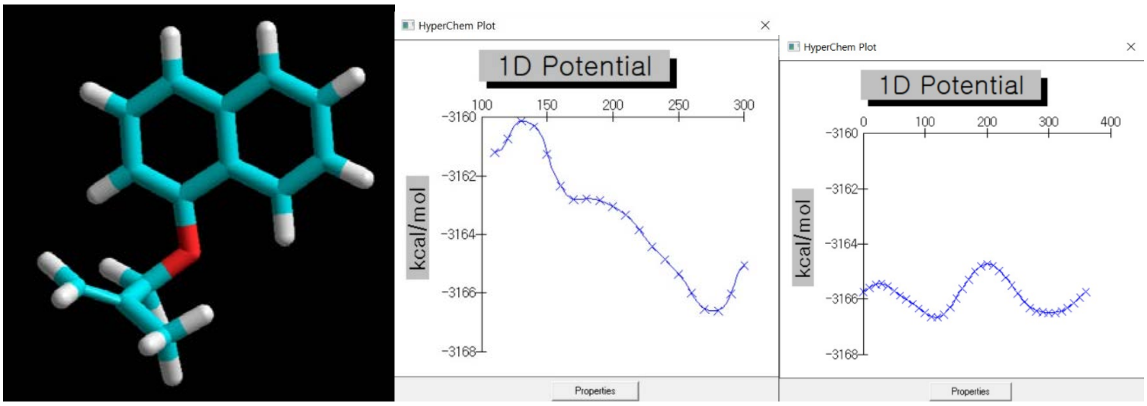

B. Allyl 2-naphthyl ether substrates

Geometry-optimized structure (left) and potential energy changes of **4b2**, depending on the C1-O-C1'-C2' (middle) and the O-C1'-C2'-C3' (right) torsional angle changes

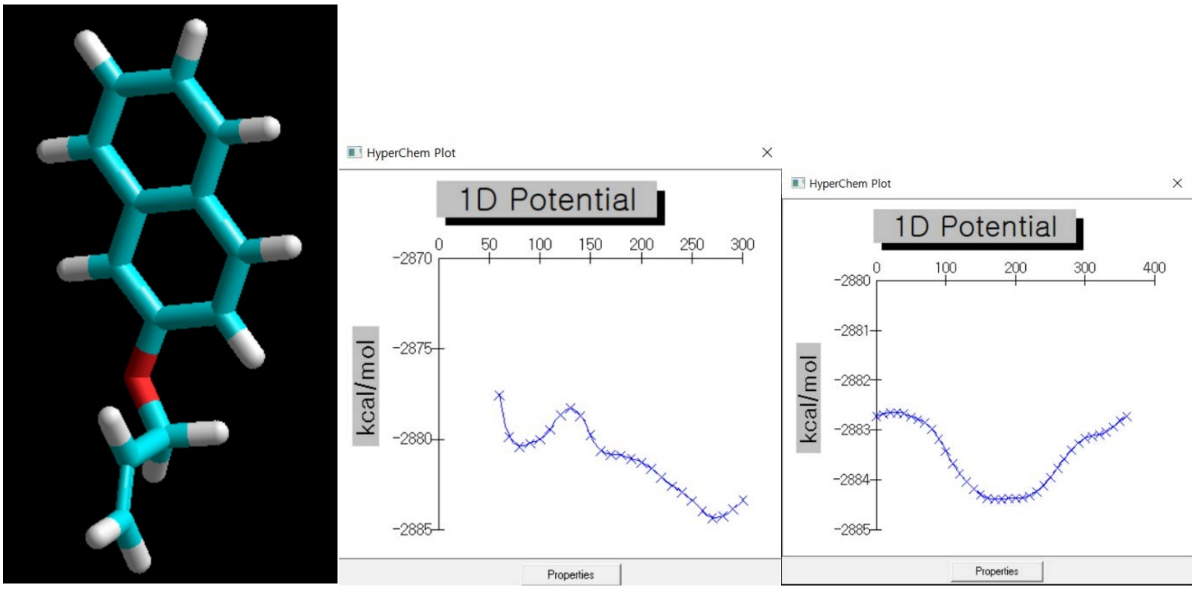

Geometry-optimized structure (left) and potential energy changes of **4c2**, depending on the C1-O-C1’-C2’ (middle) and the O-C1’-C2’-C3’ (right) torsional angle changes**.**

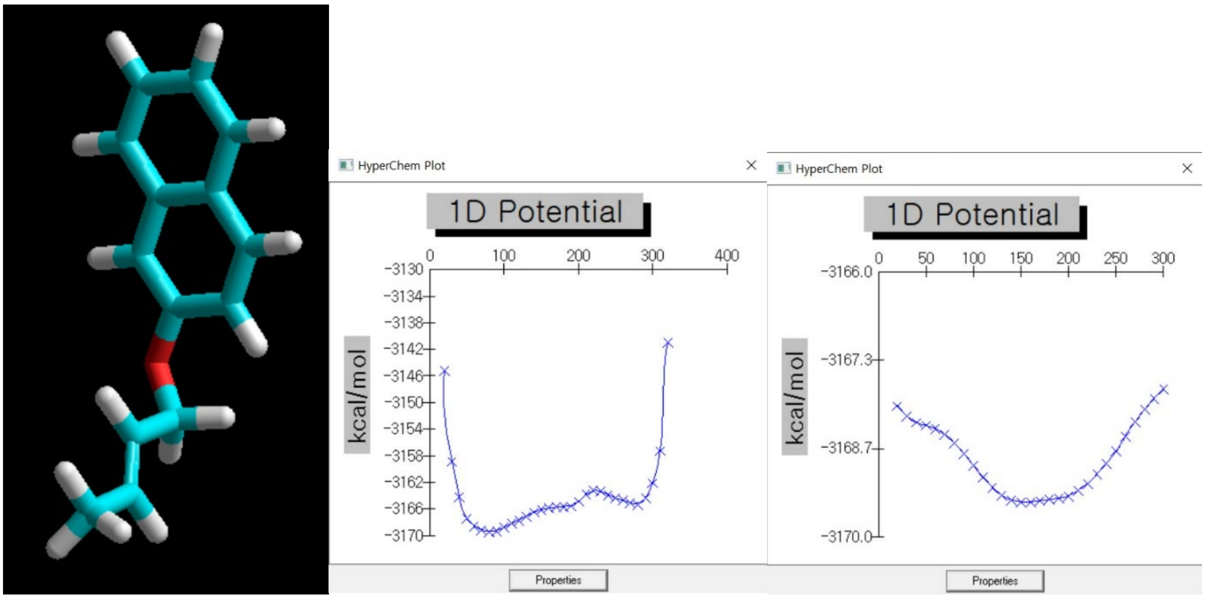

Geometry-optimized structure (left) and potential energy changes of **4d2**, depending on the C1-O-C1’-C2’ (middle) and the O-C1’-C2’-C3’ (right) torsional angle changes

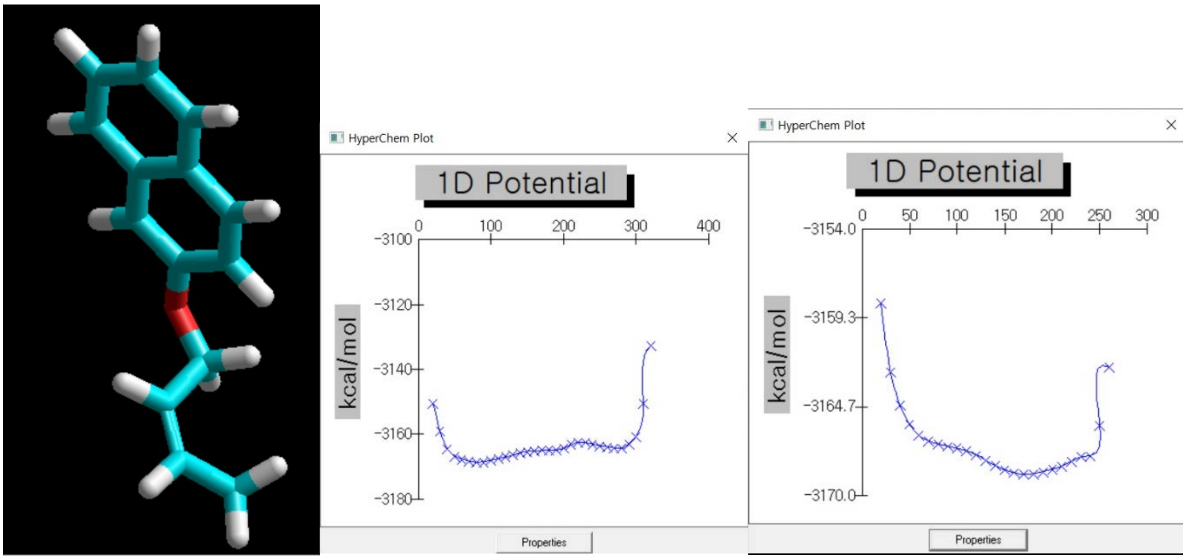

Geometry-optimized structure (left) and potential energy changes of **4e2**, depending on the C1-O-C1’-C2’ (middle) and the O-C1’-C2’-C3’ (right) torsional angle changes

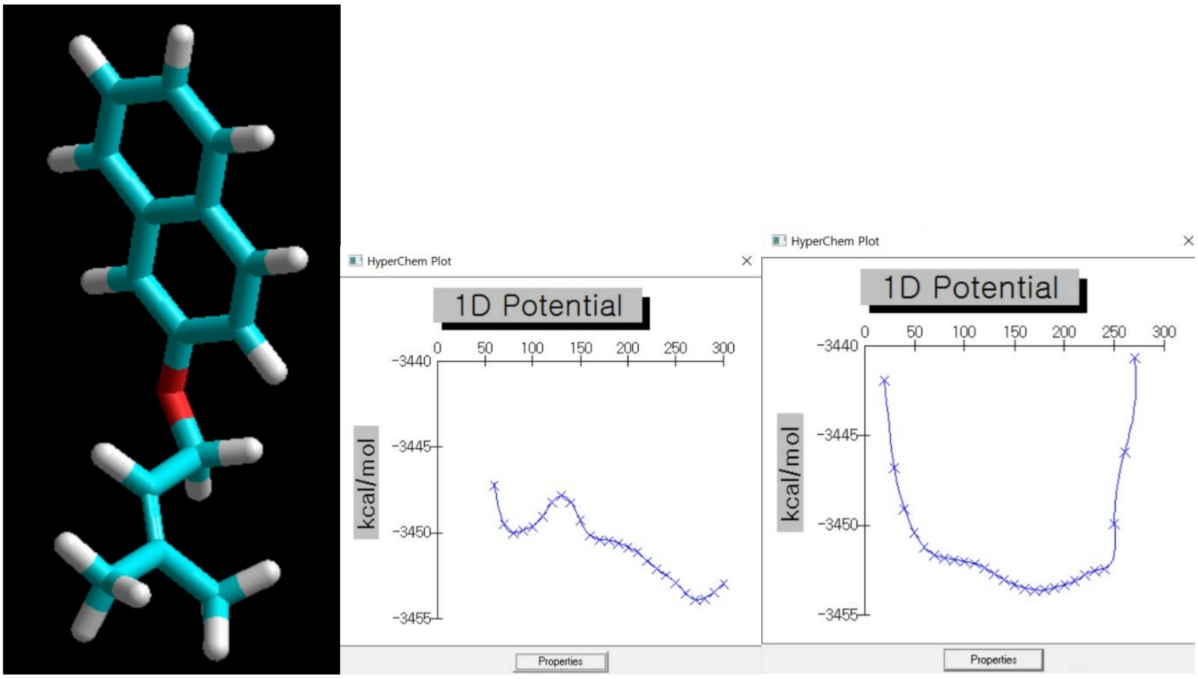

Geometry-optimized structure (left) and potential energy changes of **4f2**, depending on the C1-O-C1’-C2’ (middle) and the O-C1’-C2’-C3’ (right) torsional angle changes

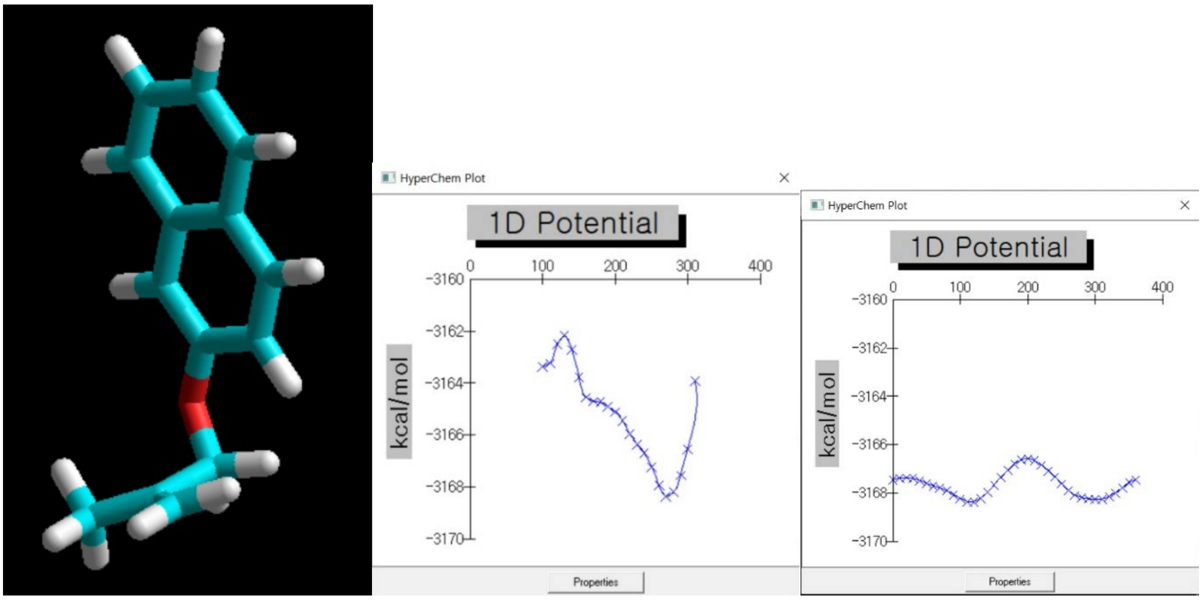

Supplement: Supplemental file 1 — Supplemental material. Download spectrum.03305-22-s0001.pdf, PDF file, 1.8 MB [file spectrum.03305-22-s0001.pdf]
